# RNA Interference: A Natural Immune System of Plants to Counteract Biotic Stressors

**DOI:** 10.3390/cells8010038

**Published:** 2019-01-10

**Authors:** Tayeb Muhammad, Fei Zhang, Yan Zhang, Yan Liang

**Affiliations:** College of Horticulture, Northwest A&F University, Yangling 712100, China; tayebmuhammad@nwsuaf.edu.cn (T.M.); feizhang@nwsuaf.edu.cn (F.Z.); zhangyan2014@nwsuaf.edu.cn (Y.Z.)

**Keywords:** RNA interference, AGOs, DCLs, RDRs, pathogens, suppressors, resistance

## Abstract

During plant-pathogen interactions, plants have to defend the living transposable elements from pathogens. In response to such elements, plants activate a variety of defense mechanisms to counteract the aggressiveness of biotic stressors. RNA interference (RNAi) is a key biological process in plants to inhibit gene expression both transcriptionally and post-transcriptionally, using three different groups of proteins to resist the virulence of pathogens. However, pathogens trigger an anti-silencing mechanism through the expression of suppressors to block host RNAi. The disruption of the silencing mechanism is a virulence strategy of pathogens to promote infection in the invaded hosts. In this review, we summarize the RNA silencing pathway, anti-silencing suppressors, and counter-defenses of plants to viral, fungal, and bacterial pathogens.

## 1. Introduction

As sessile organisms, plants have to endure a range of adverse conditions imposed by various biotic and abiotic stressors that diversely affect growth, development, and yield of plants. The occurrences and severity of the stressors may vary depending on the locality and natural environment. In particular, biotic stresses caused by the living organisms largely depend on the availability of suitable environments that activate specific organisms to infect their hosts. Among different kinds of biotic stressors that have existed since ancient time, bacteria, fungi, nematodes, and viruses are capable of causing different types of plant diseases. In response to an attacker, plants activate a specific set of defense mechanisms or related pathways, leading to either increased resistance (limits pathogen multiplication) or enhanced tolerance (reduces the effect of infection) depending on the nature of pathogen and host. RNA interference (RNAi) or RNA silencing is one of the important defense mechanisms that protects plants from pathogen infection and it controls sequence specific regulation of gene expression.

The phenomenon of RNAi was first reported in the early 1990s and it was hypothesized that introduced genes potentially co-suppressed the endogenous genes [1], but soon it was observed that homologous RNA sequences caused the suppression of internal genes [2]. The term RNA silencing was first used in a study on an animal (*Caenorhabditis elegans*) and it was observed that introduction of sense or antisense RNA caused degradation of the *par-1* gene [3]. Later, it was reported that interference of double-stranded RNAs (dsRNA) was more significant in both the injected animals and their progenies [4]. After two years, it was found that dsRNAs could convert to shorter small interfering RNAs (siRNAs) and bind to their homologous target messenger RNAs (mRNAs) [5,6]. These siRNAs also guide and instruct the multicomponent RNA-induced silencing complexes (RISCs) to cleave the specific RNAs [5]. The RNase III family enzymes were found to be involved in the cleavage of dsRNAs and could produce putative guide RNAs [7].

The proposed RNA silencing mechanism starts with the production of 20 to 26 nucleotide (nt) small RNA (sRNAs) through a series of key components, such as Dicer-like protein (DCL), Argonaute (AGO) protein, and RNA-dependent RNA polymerase (RDRs) [8,9,10]. The DCL proteins generate sRNAs from a dsRNA precursor and then incorporates into RISCs [11]. On the basis of their origin and formation, these sRNAs are divided into siRNAs or microRNAs (miRNAs). AGO proteins perform the large part of RISCs, bind the sRNAs and interact with homologous RNAs, that affect DNA methylation, endonuclease activity, or translational repression of mRNAs [12]. RDR enzymes are responsible for the synthesis of dsRNAs using single-stranded RNAs (ssRNAs) as the templates, which are then further processed by Dicer-like (DCLs) proteins and start a new round of RNA silencing [13].

In line with the diverse roles of RNAi, researchers found a number of *DCLs*, *AGOs* and *RDRs* genes in different plant species, and described their roles in plant defense mechanisms. Due to the presence of a vast number of pathogens and their threats to plants, researchers continuously study plant RNAi mechanisms as a defensive mechanism against the pathogens. The number of pathogens and RNAi pathway complexity make it difficult to understand the complete mechanism. In this review, we summarized the plant RNAi responses to various pathogens in the light of already published work and described it in a schematic way to simplify and understand the whole mechanism. Here we first, discuss the structure and nomenclature of the main three proteins of the RNAi pathway. Second, the role of these proteins and their cofactors in silencing of pathogens derived siRNAs and how they counteract the anti-silencing suppressors during plant–pathogen interaction are discussed. Finally, we summarize the recent progresses of RNAi machinery in plant immune response to viral, fungal, and bacterial diseases.

## 2. RNAi Pathway Components

### 2.1. Dicer-Like Proteins (DCLs)

DCLs are the RNase III family of endoribonucleases that contain DExD-box Helicase-C, Piwi-Argonaute-Zwille (PAZ) domain, the Domain of unknown function 283 (DUF283), RNase III, and dsRNA-binding domains (dsRBDs) [14,15]. These proteins perform the initiation stage of the RNAi mechanism, in which dsRNAs are cleaved into small RNAs 21–24 nt in length [14]. The helicases are enzymes that induce the separation of double-stranded nucleic acids, utilizing the free energy and hydrolyzing a nucleotide triphosphate to displace bound proteins [16]. A functional DExD/H-box helicase domain is also required for the efficient production of DCLs-dependent sRNAs production [17]. PAZ domain has a phosphate-binding pocket composed of arginine components that recognize the 5′ monophosphate of pre-miRNAs and are required for cleavage of dsRNAs [18,19,20]. DUF283 acts as an annealer that assists hybridization between the complementary strands of nucleic acids [21]. The function of RNase III domain is to cleave dsRNA, leaving the 2 nt overhang at 3′ end of the product [22,23]. The N- or C-termini of dsRBDs participate in the regulation of the protein nucleo-cytoplasmic distribution and also have the capacity to bind to dsRNAs [24,25]. The specific function of each domain varies depending on the Dicer protein.

Dicer proteins are conserved across the plant kingdom and up to date, various DCL proteins have been identified in different plant species, which are classified into four distinct clades. Plant DCLs produce different size of sRNAs: DCL1 produces 21 and 22 nt sRNAs from the short imperfectly hairpins RNAs, while DCL2, DCL3, and DCl4 produce 22 nt, 24 nt and 21 nt, respectively from long perfectly paired RNAs [26]. DCL1 is involved in the generation and processing of miRNAs [27,28]. Viral dsRNAs processed by DCL2 and DCL3 are required for chromatin modification and also produce heterochromatic siRNAs (hc-siRNAs) [29,30]. DCl3 is also an important element of RNA-directed DNA methylation (RdDM) pathways and it processes RDR2 generated dsRNAs into siRNAs [31]. DCL4 processes *trans*-acting small interfering RNAs (ta-siRNAs) and can be a substitute for DCL1 and DCL2 when these two are missing [32,33,34]. The product of DCLs or initiation stage is loaded into the RISCs for further processing.

### 2.2. Argonaute (AGO) Proteins

AGO proteins are highly specialized sRNA-binding modules and are considered to be the essential components of RISCs in silencing pathways [11,35]. AGO proteins perform the effector phase of silencing and the small RNAs produced in the initiation stage are loaded into AGO proteins to guide sequence-specific regulation of gene expression. Structural analysis shows that AGO proteins contain three conserved domains, such as PAZ, Middle (MID) and P-element induced wimpy (PIWI) domains. The N-terminal domain is composed of the N-terminal region and PAZ domain which facilitate the separation of small RNAs and anchor the 3′ end of the bound small RNA, respectively. The C-terminal lobe contains MID and PIWI domains, and a binding pocket at the junction of these domains that anchors the 5′ end of small RNAs [36], however, the PIWI domain can function similarly to RNase H that cleaves target mRNA [37].

Plant AGO proteins are classified into four groups and each group performs a specific function. These proteins are involved in DNA methylation and epigenetic silencing [38,39], miRNA-mediated silencing pathways [40], and stage transition of plants from the juvenile to the adult stage of growth [41]. The most important function of AGO proteins is to enhance the defense and immunity of plants through cleavage of the loaded small RNAs. Notably, AGO proteins require heat shock proteins (Hsp70–Hsp90) to chaperone and hydrolysis ATP for the loading process and conformational changes [42,43]. The size and type of the 5′ nucleotide help in the sorting of sRNAs to specific AGO proteins [35]. The less stable 5′-end pairing ds-sRNA molecule is retained within the AGO while the others are eliminated [44,45]. The action of specialized AGO proteins and small RNAs divide the plants RNAi pathways into transcriptional gene silencing (TGS) and posttranscriptional gene silencing (PTGS). AGO4, 6, and 9 have a role in TGS, while AGO1, 2, 3, 5, 7, and 10 are involved in PTGS [35]. AGO4 clade also participates in the RdDM pathway and binds the 24 nt dsRNAs produced by RDR2 and DCL3 [46].

### 2.3. RNA-Dependent RNA Polymerase (RDR) Proteins

The third phase of RNAi pathways is an amplification of silencing, performed by RDRs, which convert ssRNAs to dsRNAs and these dsRNAs are again processed by DCLs, leading to a new cycle of RNA silencing. In sRNAs, the phased and repeat-associated siRNAs depend on RDRs for biogenesis, while miRNAs and hairpin-derived sRNAs are RDRs independent RNAs. The activity of RDR was first studied in Chinese cabbage during 1971 and cDNA was isolated from tomato [47,48]. RDRs are considered as the first identified component of plants small RNAs biogenesis pathways and characterized by a unique RNA-dependent RNA polymerase catalytic domain (RdRp) [49,50]. RdRps belong to the Structural Classification of Proteins (SCOP) and initially, these proteins were identified as an enzyme in the RNA viruses that participated in the replication of the virus genome [51]. RdRps also have a role in the maintenance of genome integrity, RNA-template formation, PTGS, and defense against external RNA or DNA [52,53]. RDR proteins of eukaryotic organisms are divided into three main classes, RDRα, RDRβ, and RDRγ. RDRα exists in all three kingdoms. Animals comprise the RDRβ protein missing the RDRγ, whereas RDRγ is present in plants, and fungi contain both RDRβ and RDRγ proteins [52]. The phylogenetic analysis divides the plants RDRs into following subclasses, RDR1, 2, 3, and 6 [54,55]. In *Arabidopsis*, RDR proteins comprised six RDRs members, among them RDR-1, -2, -6 and RDRs -3, -4, -5 belong to RDRα and RDRγ clades, respectively.

RDR1, RDR2, and RDR6 are extensively studied in the plant kingdom. The general function of RDRs is further categorized to an individual member of the groups, the RDR1 is involved in the amplification of exogenous nucleic acid and defense against insects and pathogens [56,57,58]. RDR2 is an important component of the RdDM pathway and is also required for biogenesis of siRNAs and nuclear RNAi [29,59]. Interaction of RDR2 with Jumonji (JmjC) domain-containing protein (JMJ24) promotes RNA-based chromatin silencing in higher plants [60]. However, it was also reported that during sense transgenes post-transcriptional gene silencing (S-PTGS), RDR6 triggered DNA methylation of the transcribed region and RDR2 is not required for this type of methylation [61,62]. The function of RDR6–RdDM has been correctively reestablished as it triggers transposable elements (TEs) methylation and epigenetic silencing [63]. RDR6 is also involved in initial signal perception and control of miR165/166 accumulation during normal plant development [64,65]. RDR6 processes aberrant non-spliced pre-mRNAs and channels it to the RNA silencing pathway [66]. On the basis of RDRs dependent biogenesis, the sRNAs fall into two major categories, phased and repeat-associated short interfering RNAs are known RDR1/2/6-dependent sRNAs, while miRNA and other hairpin-derived sRNAs are RDRs independent sRNAs. Recently, 38 RDR1/2/6-independent sRNA-producing loci were identified in *Arabidopsis* that do not fit into any currently understood schema for plant sRNA biogenesis [67]. The most important role of plant RDRs proteins is to collaborate with other components of RNAi machinery and provide defense against invading nucleic acids. Different DCLs, AGOs, and RDRs proteins identified in plant species are listed in Table 1.

## 3. Plant RNA Silencing and Viruses

### 3.1. Plant RNA Silencing Machinery against Viruses

Viruses need a vector for transmission from one host to another, and they use host resources for their reproduction and dissemination. In plants, viruses not only produce local lesion but also a systemic infection that causes malformation, chlorosis, and stunted growth. The majority of plant viruses possess single or double stranded RNA genome except for a few families of DNA viruses. Unlike other pathogens, viruses multiply within the host cells and, hence, the RNAi pathways play a crucial role in anti-viral defense. In plants, the primary targets of the RNAi machinery are viruses with RNA genomes, which produce dsRNA intermediates during the reproduction process. DCL2 and DCL4 directly process the virus RNAs and produce 22 and 21 nt siRNAs respectively, which is further loaded to the AGO1 and AGO2 complex for cleavage [84]. In the case of DNA viruses, dsRNA phase is absent in their replication cycle, and RDR6 and Suppressor of gene silencing 3 (SGS3) are required for RNA silencing [85]. DNA viruses also require all four DCLs proteins to produce 21, 22, and 24 nt siRNAs in the host cells [86].

Virus multiplication increases accumulation of virus-derived siRNAs (vsiRNAs) during infection, and many studies have shown that virus inoculation induces the transcription level of various RNAi genes in different plant species. For example, Cucumber mosaic virus (CMV) enhances RDR1, RDR2, and RDR3 in *Salvia miltiorrhiza* [78], Tomato yellow leaf curl virus (TYLCV) up-regulates most of the tomato DCLs, AGOs, and RDRs [55], CMV, Potato virus Y (PVY) and Tobacco mosaic virus (TMV) infections up-regulate *CaDCL2*, *CaDCL4*, *CaAGO2*, and *CaAGO10b* in *Capsicum annuum* [70]. Similarly, CMV infection in *Arabidopsis* results in the accumulation of CMV-vsiRNAs of 21-, 22-, and 24 nt, wild-type and *dcl1*, *2*, and *3* single mutants produce 21 nt species after infection, which is abolished in the *dcl4* mutant [87]. Whereas, *dcl2*-*1* and *dcl3*-*1* mutants lack 22 nt and 24 nt vsiRNAs after CMV infection [88], revealing that CMV infection requires DCL2, DCL3, and DCL4 for the accumulation of various classes of vsiRNAs that are needed for antiviral silencing. DCL2 and DCL4 are crucial against RNA viruses, and viral RNA accumulation and systemic infection are more effective in both DCL2 and DCL4 inactive plants [89,90,91]. DCL2 is essential to promote the cell-to-cell spread of virus-induced RNA silencing, while DCL4 participates in cell-autonomous intracellular silencing and inhibits intercellular silencing, the DCL2 and its 22 nt vsiRNA may also substitute for the DCL4 when it is absent or suppressed by viruses [92,93]. The 24 nt siRNA produced by DCL3 is not enough against invading RNA, and, thus, the production of 21- and 22 nt species is needed for an effective silencing process. However, accumulation of the 24 nt transgene-derived siRNAs (t-siRNAs) are associated with plant immunity against crinivirus [94], which shows that DCL3 play minor role in antiviral defense against RNA viruses.

In case of DNA viruses, the DCL3 and its cognate 24 nt vsiRNAs are associated with RdDM to protect the plant from infection [95,96]. In addition to RdDM, hyponastic leaves 1 (HYL1) family proteins and Hua enhancer 1 (HEN1) are involved in DCLs induced biogenesis of DNA virus vsiRNAs [86]. HEN1 also methylates all endogenous sRNAs and protects them from uridylation activity [97]. It was reported that *Hen1* mutants were more susceptible to CMV and TCV virus infections [92,98]. In the Cabbage leaf curl virus (CaLCuV), DCLs produce different classes of virus siRNAs, but the dsRNA precursors for these vsiRNAs are produced by RDRs independent pathways and do not require RNA polymerases IV and V (Pol IV and Pol V) [99]. These are generated by RNA polymerase II through transcription of viral DNA [96]. Like RNA viruses, DCLs also function in the antiviral mechanism of DNA viruses. TYLCV inoculation induces the expression of *SlDCLs* and silencing of the *SlDCL2*/*SlDCL4* increases the sensitivity of tomato to TYLCV infection [100], confirming that 21 and 22 nt siRNA production is also required for the plant–DNA viruses silencing mechanism.

Plants also contain special double-stranded RNA binding (DRB) proteins that promote DCLs for the precise production of small RNAs. DRB proteins are non-catalytic factors having double-stranded RNA binding motif (dsRBM). In *Arabidopsis*, the *AtDRB2*, *AtDRB3*, and *AtDRB5* are involved in the miRNA biogenesis pathway [101,102], and *AtDRB3* and *AtDCL3* together affect the methylation of the viral genome [103]. Interactions of DCL1 and DRB1 are required for the efficient production and loading of miRNA, while DRB2 binds DCL1 for miRNA biogenesis and improves silencing action [104,105]. DRB2 is also involved in the recruitment of repressing epigenetic factors that fine-tune the transcription at targeted loci, and the loss of function of DRB2 accumulates a higher amount of RNA polymerase IV-dependent siRNAs, suggesting that the transposable element transcript would be converted by the RdDM pathway [106,107]. Similarly, DRB4 facilitates the DCL4 activity for biogenesis of miRNAs and antiviral silencing in plants. DRB4 and DCL4 collectively participate in Turnip yellow mosaic virus (TYMV)-derived siRNA production and antiviral responses [108], but in the absence of DRB4, other members of this family might assist DCL4 in the biogenesis of 21 nt vsiRNA [109]. However, the interactions of some DRB members (DRB7.1) with DRB4 or DRB complex antagonize the production of siRNAs and RNase III activity in plants [110]. In contrast, the DRB7.2 directly interacts with DRB4 and participates in the epigenetically activated siRNAs pathway [111]. Recently, it was reported that the interaction of conserved Forkhead-associated (FHA) domain-containing protein DAWDLE (DDL) with DCLs is required for the biogenesis of siRNAs. The DDL particularly interacts with DCL3 and improves its activity [112]. Moreover, DDL has also been shown to be required for development and immunity [113,114].

**Figure 1:**
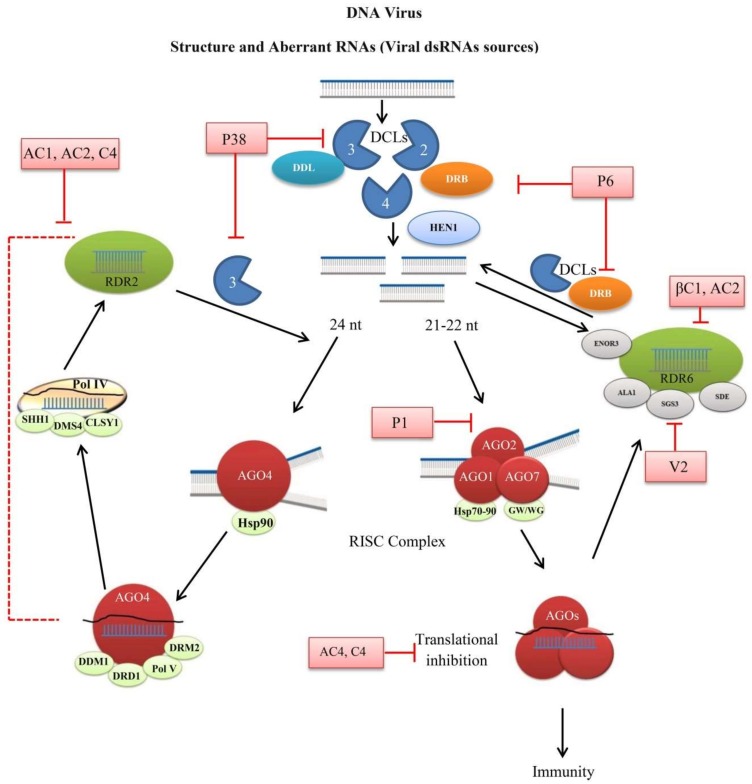
Schematic diagram of plant RNA silencing against DNA viruses. Plant Dicer-like proteins (DCLs) generate 21, 22 and 24 nt siRNAs from viral dsRNA. The 21 and 22 nt siRNAs are incorporated into Argonaute (AGOs) containing RNA-induced silencing complex (RISC) for silencing or translational inhibition. The RISC product may enter into the amplification line and produce secondary small interfering RNAs (siRNAs) through the actions of RNA-dependent RNA polymerases (RDRs) and cofactors. While, the 24 nt siRNAs are methylated through RNA-directed DNA methylation (RdDM) pathway including AGO4, RNA polymerase (Pol IV and V), RDR2 and finally processed by DCl3. Viral silencing suppressors (VSRs) block the RNAi mechanism by inhibiting the function of various components (pink box). Double-stranded RNA binding (DRB) proteins, Hua enhancer 1 (HEN1), DAWDLE (DDL), Heat shock proteins (Hsp), Silencing defectives (SDE), Suppressor of gene silencing 3 (SGS3), Aminophospholipid ATPase 1 (ALA1) and Enhancer of RDR-3 (ENOR3) proteins. Glycine/Tryptophane (GW), DNA-dependent RNA polymerases (Pol IV and V), Domain rearranged methyltransferase 2 (DRM2), Defective in RNA-directed DNA methylation 1 (DRD1), Deficient in DNA methylation 1 (DDM1), Classy 1 (CLSY1), Defective in meristem silencing 3 (DMS3), and Sawadee homeodomain homolog 1 (SHH1).

The siRNAs produced by DCLs are incorporated into AGOs, which is an important downstream step for silencing of external nucleic acid. In plants, various AGOs were identified, and functions of few members against multiple viruses were revealed. Genetic analysis of the *Arabidopsis* AGO proteins showed that only AGO1, 2, and 7 are involved in the virus resistance and have a role in RNA silencing and restriction of the virus [115,116]. Among them, AGO1 is considered as the first activation layer and AGO2 as a second defense layer to restrict virus accumulation after AGO1 is suppressed by the virus suppressor. The activation of the second defense also indicates that the first layer is lost due to the suppression of AGO2 by AGO1 through miR403 [117]. Similarly, AGO5 performs an antiviral role in the absence of AGO2. However, both have the ability to bind vsiRNAs. The *ago2 ago5* double mutants are more susceptible to the virus than single mutant [118,119]. AGO1 has the capability to target compact structure viral RNAs, while AGO7 favors less structured RNAs and both are involved in the silencing of Turnip crinkle virus (TCV) RNAs [120]. *Ago1*, *ago2*, and *ago7* (knockdown) mutants show increased hyper-susceptibility to CMV, TCV, TMV, potato virus X (PVX) and ringspot virus [117,120,121,122,123,124]. Similarly, plants require AGO proteins (AGO4, AGO6, AGO9) for systemic silencing where AGO4 plays a crucial role in maintaining or activating the expression of stress-responsive genes via chromatin modification or preventing cryptic transcription [125,126]. This clade is localized in the nucleus and the 24 nt siRNAs associated to AGO4 are dependent on DNA-dependent RNA polymerase IV, RDR2, and DCL3, showing the binding of AGO4 to long coding RNAs [39,127,128] (Figure 1). In case of *Plantago asiatica* mosaic virus (PlAMV), the virus changes the AGO4 localization from nuclear to cytoplasmic distribution and AGO4 target virus RNAs are independent of RdDM components (DCL3, Pol IV, and Pol V) [129]. This independent class of siRNAs mainly originates from transgenes, transposons, and intergenic sequences [130].

Previous studies reported that *ago4* mutants are more susceptible to Tobacco rattle virus (TRV) and Beet curly top virus (BCTV) [131,132,133]. AGO4 is also involved in host transcriptional response and resistance against CMV and PVX viruses [134,135]. Recently, researchers have identified many new members of the AGO proteins in different plant species, but their functions still remain unknown. Therefore, research in the current direction may be helpful to better understand the effector phase of silencing.

The host RDRs proteins use the viral primary siRNA molecules as a primer and convert them into long dsRNAs, leading to the amplification of siRNA signals. RDR1, RDR2, and RDR6 are considered important members that act in the various biological processes of RNA silencing. Recently, it was found that Tobacco mosaic virus-coat protein (TMV-CP) accumulation was less in the *CaRDR1* overexpressing tobacco lines following TMV infection. Furthermore, TMV inoculation enhanced transcript levels of *AGOs* and *DCls* genes in the *RDR1* expressing lines [136]. Similarly, *MdRDR1* transgenic tobacco plants enhanced resistance against TMV, Sunn hemp mosaic virus and Turnip vein-clearing virus [137,138]. In the case of CMV, the siRNAs production depends on the RDR1, and these secondary vsiRNAs are important for antiviral silencing [88]. Silencing of *RDR1* or knockout *rdr1* mutants result in a higher level of virus RNA and reduces resistance in pepper, *Arabidopsis*, maize, and tomato plants [136,139,140,141], however, the resistance of potato to PVX and PVY is not compromised [142]. As we mentioned, RDR2 protein along with DCL3 is involved in the production of 24 nt siRNAs, and loss of RDR2 function causes extensive changes in the expression of genes, transposons and 24 nt sRNAs [61]. This protein is considered as the main component of the RdDM pathway and required for cell-to-cell silencing and the reception of the long-distance mRNA silencing signal [49,64]. RDR2 is also involved in TGS pathway and in antiviral defense [50,143,144]. Loss of RDR2 function results in the up-regulation of certain elements that are members of *Ty3*/Gypsy-like super-family of retrotransposons and these elements are transcriptionally silenced via the RdDM pathway. *Ty-1* and *Ty-3* are resistance allele of wild-type tomato against the TYLCV virus, these alleles encode the DFDGD motif and belong to the RDRγ clade [145,146]. It is believed that various *Ty* genes are involved in RNA silencing and viral genomes cytosine methylation [145,147,148]. *Ty-3* and *Ty-4* significantly lower the Tomato mottle virus (ToMoV) disease severity, while *Ty-6* and *Ty-5* provide resistance against TYLCV [149]. Genetic analysis of interspecific crossing between *Solanum habrochaites* and cultivated tomato show that a nucleotide-binding domain and leucine-rich repeat (NB-LRR) containing gene, *TYNBS1*, is synonymous with the *Ty-2* gene and strictly co-segregated with TYLCV resistance [150]. Similarly, the *Ty-1* and *Ty-3* markers were detected in the TYLCV resistant tomato lines, but not in the susceptible lines [151].

**Figure 2:**
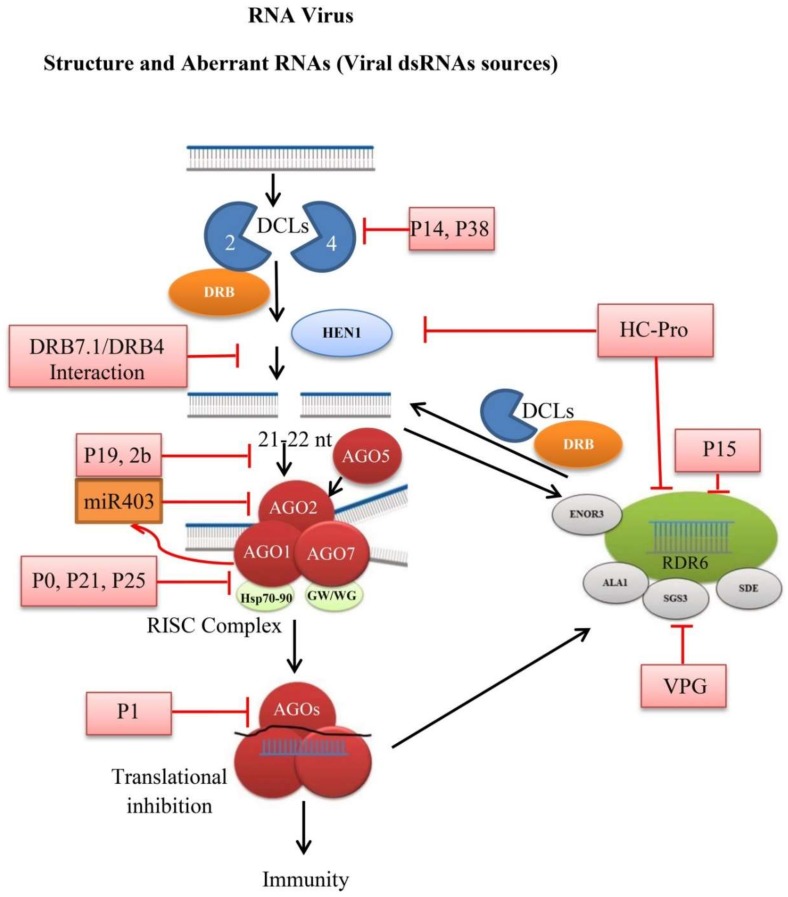
Schematic diagram of plant RNA silencing against RNA viruses. DCLs generate 21–22 nt siRNAs from viral dsRNA. The siRNAs are incorporated into AGOs containing RISC for silencing or translational inhibition. The RISC product may enter the amplification line and produce secondary siRNAs through the actions of RNA-dependent RDRs and cofactors. VSRs can block the RNAi mechanism by inhibiting the function of various components (pink box). AGO2 suppression by AGO1 through miRNA (orange box). DRB proteins, HEN1, Hsp, SDE, SGS3, ALA1, and ENOR3 proteins.

The RDR2 also competes with RDR6 for RNA substrate and antagonizes RDR6, because RDR2 is functionally more active in the presence of counteractive siRNA; as a result, *rdr2* mutants show increased efficiency of sense transgenes post-transcriptional gene silencing (S-PTGS) [62]. As systemic and intracellular PTGS requires RDR6 that restricts pathogen at both the systemic and single cell level [152]. *NbRDR1* expression up-regulates in *RDR6*-silenced plants infected with CMV-2b non-expression mutant, and RDR1 synergistically functions with RDR6 to facilitate immune responses [153]. RDR6 activity is assisted by protein cofactors Suppressor of gene silencing 3 (SGS3) and Silencing defectives (SDE3 and SDE5) [50,154,155,156]. Due to RNA silencing, the apical meristem of plants shows resistance to viral infection, but in case of *Arabidopsis rdr6* mutant, the CMV wild and mutant type can cause severe symptoms with minimum antiviral activities in the apical meristem [157]. Recently it was found that membrane-localized flippase Aminophospholipid ATPase 1 (ALA1) and Enhancer of RDR-3 (ENOR3) proteins cooperate with RDR6 to promote antiviral immunity. *Arabidopsis* double mutants *ala1-2 rdr6* and *enor3-1 rdr6* exhibit severe symptoms after inoculation with CMV. Both ALA1 and ENOR3 inhibit the accumulation of CMV and functions additively with RDR1 and RDR6 to mediate plant immunity [158,159]. Tobacco RDR6 expressing lines show enhanced resistance to PVY while on the other hand, its silencing results in the accumulation of viral RNAs and increased susceptibility to the Chinese wheat mosaic virus (CWMV), Rice dwarf phytoreo virus (RDPV) and Rice stripe virus (RSV) [160,161,162,163]. In some viral infections, the accumulated viral siRNAs are RDRs independent and act as a poor template for production of secondary siRNAs. For instance, the accumulated vsiRNAs of Pelargonium line pattern virus (PLPV) and CaLCuV are not associated with the RDRs activity [96,164]. Taken together, RNAi mechanism is an important plant defense mechanism that mimics viral pathogens, but the research body is relatively less regarding the other members of these gene families and, therefore, more study is needed to unravel the functions. The reported function of various components of plants RNAi pathway against the RNA viruses and suppression by silencing suppressors is presented in Figure 2.

### 3.2. Viral Suppressors Block Plant RNA Silencing

To neutralize the effect of RNAi, well-adapted plant viruses have developed viral suppressors of RNA silencing (VSRs), which largely attenuate plant defense against such type of viruses [165]. It is suggested that the VSRs are essential for successful viral infection and most of the viruses contain at least one VSR that blocks the RNA silencing at any step. In CMV infection, the accumulation of various viral siRNAs produced by DCL1, DCL2, and DCL3 is reduced by the expression of 2b suppressor [88]. Similarly, the P6 of Cauliflower mosaic virus (CaMV) interacts with DRB4, an important partner of DCL4 and affects the viral siRNA processing [166]. In Turnip crinkle virus infection, P38 suppresses DCLs, but its main target is DCL4, as DCL4 produces silencing signal to restrict virus exit from vascular bundles and P38 inhibits this signaling [167]. In plants, Glycine/Tryptophane (GW)-containing proteins are required for miRNA and siRNA RISC function. TCV P38 and Sweet potato mild mottle virus (SPMMV) encode GW P1 proteins that interact with cellular AGOs and isolate AGO1 from associated siRNA and miRNA effector complexes preventing loading and RNA silencing [168,169]. CMV 2b and TRV 16K suppressors restrict the initial formation of miRNA- and siRNA-guided RISCs and bind to AGO4 to prevent target RNA cleavage, inhibit AGO4 slicer activity, and the methylation of numerous loci [133,135]. Moreover, 2b and P0 proteins inhibit the RISC activity through interaction with the PAZ domain of AGOs and directing its degradation [170,171,172]. The widespread distribution of P0 in host cells hijacks the F-box containing complex or SKP1-CUL1-F-box (SCF) machinery to stop silencing by destabilizing AGO and results in severe disease infections [172,173]. In SPMMV, P1 suppressors block target RNA binding to AGO1 and the zinc finger domain of P1 performs the suppression activity [174]. The Pelargonium flower break virus coat protein (PFBV-CP) and Tomato bushy stunt virus (TBSV) P19 suppressor also prevent the incorporation of the viral siRNAs into the RISC complex [175,176,177], however, P19 specifically impairs loading into AGO1 but not AGO2 [178], and TBSV *p19* mutant is highly susceptible to RNA silencing [179,180]. Citrus tristeza virus (CTV) P20 and P23 suppress salicylic acid (SA) and the RNA silencing defense pathways of orange plants [181,182]. The existence of more suppressor proteins in one virus seems to be lead in targeting various host defense pathways at the cellular level for more successful infection. Olive mild mosaic virus coat protein (OMMV-CP) and P6 show suppressor activity with complementary manner and silencing of both suppressors result in significant reduction of viral accumulation and symptoms compared to single silencing [183].

Virus movement and replication in the host cell need to suppress RDRs activities to stop the silencing amplification and distinct signaling. In tobacco plants, symptom development after inoculation of two different begomoviruses depends on the interaction of virus AV2-encoded pre-coat protein and host RDR1. The pathogen AV2 represses the RDR1-mediated silencing for the establishment of disease symptoms in the hosts [184]. Helper component proteinase (Hc-Pro), P19, and P38 altered accumulation and reduce primary siRNAs level to suppress the RDR6-directed transitive RNA silencing. Similarly, P19, P38, and P15 constrain the RDR6-dependent *cis-* and *trans*-acting inhibitory effects that normally restrict the accumulation of PVX in *Arabidopsis* [185]. The V2 protein of TYLCV suppresses the RNA silencing pathway by either directly interacting with RDR6 cofactor (SGS3) or enhancing the accumulation of Calmodulin-like proteins (CaM) that cause SGS3 degradation through autophagic pathways [186,187]. Notably, a mutation in amino acid on position 71 affects the self-interaction, aggregation, and pathogenicity of V2 [188]. Tomato spotted wilt virus NSs proteins can replace HC-Pro–deficient potyvirus function and favor the development of local and systemic symptoms [177]. Likewise, mungbean yellow mosaic Indian virus (MYMIV) AC2 protein suppresses RNA silencing mechanism by interacting with the host RDR6 protein [189].

In addition to the suppression of host RNAi machinery, certain suppressors are involved in virus replication and movement, and they need a host factor for successful infections. Cysteine-rich protein (CRP), 6K1 and 29K are important elements that contribute to RNA silencing suppression in the context of virus replication at early stage of infection, whereas Triple gene block protein 1 (TGBp1), HC-Pro, P3, and P3N-PIPO are required for viral cell to cell and long-distance movement [190,191,192,193,194]. RNA viruses (positive-strand) enhance the biogenesis of virus factories bound to the cytoplasmic membrane, and upon infection, the 6K2 protein-induced replication vesicles form chloroplast-6K2 complexes by targeting the chloroplast for replication [195]. The two host proteins Syp71 and Vap27-1 are associated with the chloroplast-bound 6K2 complex. Silencing of these factors showed that Syp71 but not Vap27-1 is essential for mediating the fusion of the virus-induced vesicles with chloroplasts during virus infection [196]. It was observed that two viral suppressors, carmovirus P38 and potyvirus HC-Pro require ethylene-inducible host transcription factor RAV2 to divert host defenses toward responses and block RNA silencing [197]. Furthermore, the Viral genome-linked protein (VPg) and HC-Pro suppressors interact with Decapping protein 2 (DCP2) and Exoribonuclease 4 (XRN4) respectively, both are key proteins of cytoplasmic 5′-3′ RNA decay gene-encoded pathway (5′ RDGs). The interaction results in the suppression of RNA silencing and promotes viral infection [198]. In DNA viruses, suppressors betaC1 (βC1) and AL2 proteins require host CaM for complete function. The βC1 expresses the CaM to repress the *RDR6* expression and stop the production of secondary siRNAs, while AL2 influences defense response genes through CaM and eventually blocks the RNA silencing [199,200].

## 4. Plant RNA Silencing Machinery against Fungi

Fungi are eukaryotic organisms, consisting of more than one million species and are considered as one of the most important groups of plant pathogens causing huge yield losses in various economic crops. Fungal pathogens penetrate the host through a natural opening, wound or through the specialized hyphal structures. The gene organization, cellular structures, and metabolic mechanisms of the fungus are similar to those of other higher eukaryotic organisms such as animals and plants. Beside the pathogenicity, certain fungal species also act as vectors for the transmission of different viruses. Similar to other eukaryotes, fungi also contain basic RNAi components to perform antiviral activities. The presence of RNAi components has been described in many fungal species [201,202,203,204], and these components have similar functions of antiviral activity like in higher eukaryotic organisms [205,206,207,208].

**Figure 3:**
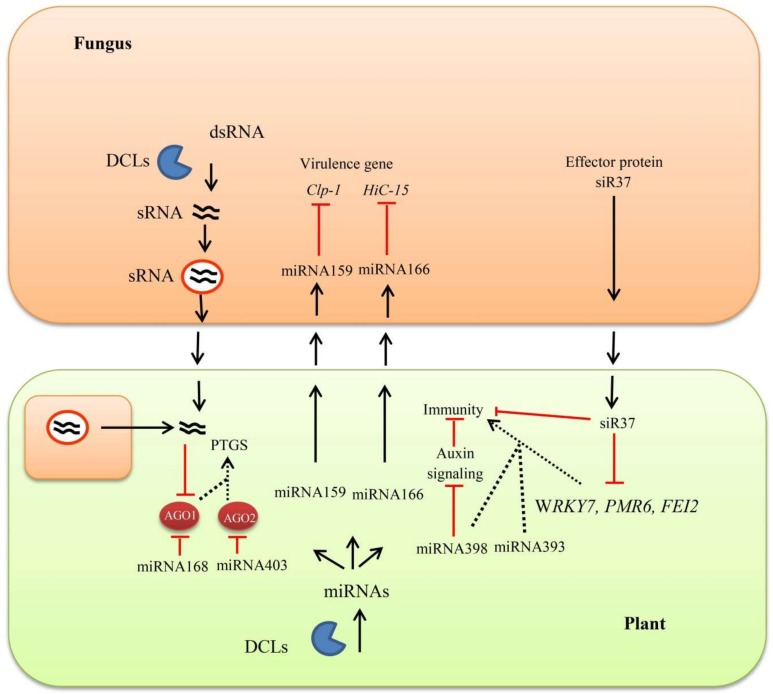
Role of RNAi in plant–fungal pathogen interaction. Plants activate biogenesis of various small RNAs or microRNAs (miRNAs) that enhance or inhibit certain signaling relating to the resistance or susceptibility against fungal pathogens. Some miRNAs are delivered to pathogens through cross-kingdom RNAi to perform silencing or inhibit virulence related genes of the interacting pathogens. The fungal pathogens also deliver certain effector proteins that hijack the RNAi components or suppress host defense related genes. Posttranscriptional gene silencing (PTGs), isotrichodermin C-15 hydroxylase (HiC-15), and Ca2^+^-dependent cysteine protease (Clp-1).

Plant and plant fungal pathogen share similar RNAi machinery, which on the one hand is used by the hosts to defend pathogens, while on the other hand, is utilized by the invading organism for growth, development, and pathogenesis. Fungal DCLs produce sRNAs that bind to the plant AGO1 proteins to hijack the RNAi machinery and silence host immunity (Figure 3). In the case of *Botrytis cinerea*, *dcl1 dcl2* double mutants show reduced virulence due to lack of plant immunity suppressing sRNAs produced by active *DCls* genes [209]. The *VmAGO2* of *Valsa mali* is critical for pathogen virulence which causes disease on apple leaves and twigs [210]. *Colletotrichum higginsianum* mutant study reveals that various DCLs, AGOs, and RDRs have no effect on the vegetative growth of fungus, however, *Δdcl1*, *Δdcl1Δdcl2* double mutants, and *Δago1* mutants exhibit severe defects in conidia formation and morphology [211]. DCL double mutants of *Colletotrichum gloeosporioides* also lack conidiation and penetration capability to the host plant, leading to poor manipulation of the host immunity [212]. Conidia formation and the entrance of fungal pathogen to host cells are an important step in disease development and countering the host immunity. The above report described the importance of the RNAi components that are involved in the virulence nature of plant fungal pathogens.

The direct involvement and differential expression of the plant RNAi components showed contradictory results against fungal pathogens in different host plants. *Arabidopsis* mutants *dcl4*, *ago1*, *ago2*, *ago7*, *ago9*, *rdr1*, *rdr2*, *rdr6*, *sgs1*, *sgs2*, *sgs3*, and *nrpd1a* are more susceptible and display enhanced symptoms upon *Verticillium dahlia*, *Sclerotinia sclerotiorum*, and *Phytophthora species* infections [69,213,214,215]. However, silencing of *DCL1* enhances resistance to *Magnaporthe oryzae* and *S. sclerotiorum* in rice and *Arabidopsis*, respectively [69,216]. Such type of DCL1 interaction may be due to it being involved in the biogenesis of miRNA, and upon the infection of virulent fungus, the DCL1 reduces the accumulation of certain miRNAs, that induce the down-regulation of pathogen resistance genes [216]. It was previously found that the suppression of DCL1 resulted in the reduction of miRNA accumulation [217]. Similarly, knockout of AGO1 protein enhances resistance to *V. dahlia* and *Verticillium longisporum* [213,218]. The fungal virulence mechanism involves the AGO1 protein function through suppression of miR168 expression, as miRNA168 targets the RNA slicer enzyme of miRNAs pathways and the variation in expression of miRNA168-AGO1 plays an important role in plant–fungus interaction [218,219]. In tomato, the expression of two miRNAs suppresses the nucleotide-binding (NB) resistance (*R*) genes that have a function in plant immunity against *Fusarium oxysporum* and the potential resistance in the susceptible materials is insufficiently activated due to the action of these miRNAs [220]. Notably, miRNAs are non-coding sRNAs generated by DCLs from hairpin ssRNAs precursors, which target transcripts to regulate gene expression post-transcriptionally for cleavage or translational repression [221]. Large numbers of miRNAs were reported to be functionally involved in fungal stress response. In *Brassica napus*, the *S. sclerotiorum* differentially expresses 68 miRNAs between the inoculated and un-inoculated leaves. A set of these miRNAs target nitric oxide- and reactive oxygen species-related and nucleotide-binding domain and leucine-rich repeat (NB-LRR) resistance (*R*) genes, and these genes are involved in plant defense response to various pathogens [214]. miR160a, miR396a, miR398b, miR482, miR1444, miR2118, and miR7695 are few examples of plants’ microRNAs that are involved in gene regulation and immunity against fungal pathogens [222,223,224,225,226].

In plant–pathogen interaction, a diverse class of sRNAs contributes to host immunity through gene silencing but the pathogen-derived sRNAs also trigger pathogen virulence. Sometimes this influence can extend to other kingdoms and regulates gene silencing in the interacting organism; such type of interaction is called cross-kingdom RNAi. In cross-kingdom RNAi, the silencing signals translocate from both sides of the interacting organisms and perform gene silencing of the opponents. It was observed that *Arabidopsis* secreted extracellular sRNAs deliver vesicles at infection sites and are taken up by *Botrytis cinerea* cells [227]. Cotton plants induce biogenesis of two specific miRNAs upon *Verticillium dahliae* infection and export them to the fungal cells for silencing. Both miR159 and miR166 target different virulence-related genes of the fungal pathogens and confer disease resistance [228]. The secretion of these extracellular vesicles (EV) not only enhanced during biotic and abiotic stresses but also in response to hormonal treatment and contributes in plant innate immunity [229]. Using the cross-kingdom interaction, *B. cinerea* delivers sRNA effectors proteins into the host cells and down-regulates host RNAi and defense-related genes [230]. To protect the plant from pathogen sRNAs silencing, an alternative method of host-induced gene silencing (HIGS) is used to trigger plant immunity. In HIGS, exogenous artificial RNAi signals are expressed to target pathogen mRNAs for gene suppression and enhanced crop resistance. This approach is successively reported in plant–fungus interaction. In tobacco plants, interfering intron-containing hairpin RNA constructs transform to target chitin synthase (*chs*) gene, an important component of fungal cell wall chitin synthesis. RNA constructs silence the *chs* genes in transformed plants and provide efficient resistance against *S. sclerotiorum* [231]. Similarly, HIGS of an essential fungal chitin synthase gene (*Chs3b*) enhances resistance to *Fusarium* blight in wheat [232]. Suppression of *V. dahliae* virulence factors (*Ave1*, *Sge1*, and *NLP1*) through the HIGS approach reduces the susceptibility of *Arabidopsis* and tomato to *Verticillium* wilt [233]. Transgenic wheat plants cause severe hyphal cell wall defects in *Fusarium culmorum* via RNAi hairpin silencing construct against the β-1, 3-glucan synthase gene *FcGls1* and show enhanced resistance in leaf and spike [234]. Studies have revealed the similar role of HIGS in suppressing diseases caused by various groups of fungi [235,236,237,238].

## 5. Plant RNA Silencing Machinery against Bacteria

Plants and bacteria interact with each other in a variety of ways. The interactions may be useful, harmful or neutral. The deleterious interactions lead to the development of disease on the host plants, and the rapid expansion of the bacterial diseases make it difficult to control. Pathogenic bacteria restrict plant development by using their virulence factors delivered via multiple processes like the production of phytohormones, quorum sensing, siderophores, exopolysaccharides, and the Type III secretion system (T3SS) [239,240,241,242,243,244]. Plant primary defense mechanisms start after the recognition of bacterial translational or flagellum components. To counteract the pathogen-associated molecular pattern (PAMP)-triggered immunity (PTI) system, bacteria deliver various effectors into the host cells and alter the transcriptome and proteome of the cell to make it susceptible to the pathogen. The plant triggers the second defense layer in the form of effector-triggered immunity (ETI) after the recognition of pathogen effectors. Several endogenous siRNAs and miRNAs were reported to fine-tune PTI and ETI responses [245,246]. PTI and ETI mainly target the bacterial iron-related sigma factors to disturb bacterial iron metabolism, as high expression of these factors provides tolerance to bacteria against plant immunity [247].

**Figure 4:**
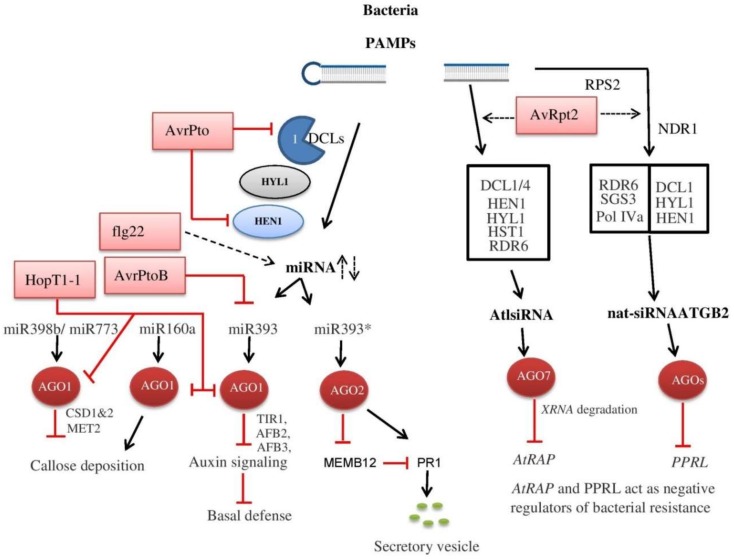
Role of RNAi in plant–bacterial interaction. Upon bacterial infection, plants detect pathogen-associated molecular patterns (PAMP) and control the accumulation of various siRNA or miRNAs through RNAi machinery. These sRNAs either enhance the defense related signals and resistance genes or silence certain genes that negatively regulate plant immunity. In response to the PAMP-triggered immunity (PTI) system, bacteria produce certain effectors that suppress host RNAi mechanism (pink box). Hasty1 (HSTY), Resistance protein 2 (RPS2), Non-race specific disease resistance protein (NDR1), Pentatricopeptide repeats protein-like (PPRL), RNA binding domain abundant in apicomplexans (RAP), MEMB12 (Membrin), Pathogenesis-related protein (PR1), Transport inhibitor response 1 (TIR 1), Auxin signaling F-Box proteins 2 and 3 (AFB2&3), Copper superoxide dismutases 1 and 2 (CSD1&2), Methyltransferase (MET).

The virulent *Pseudomonas syringae* contains type III secretion effector that suppresses the activity, stability, and biogenesis of pathogen-responsive miRNAs. *P. syringae* pv. *tomato* DC3000 *hrcC* mutant, a non-virulent form of bacteria due to lack of T3SS is unable to suppress PTI in wild-type *Arabidopsis* but enhances bacterial growth in the miRNA deficient *dcl1-9* and *hen1-1* mutants [248]. Similarly, *dcl1-9* and *hen1-1* mutants show similar symptoms of bacterial disease upon inoculation of the nonpathogenic *Escherichia coli* W3110 and *Pseudomonas fluorescens* Pf-5 strains [248]. In contrast, the *dcl1* and *hen1* mutants show enhanced defense against *Agrobacterium tumefaciens* induced gall formation and conversely, *rdr6* mutants are significantly susceptible to *A. tumefaciens* infection [249], revealing that a successful infection needs a strong anti-silencing mechanism to inhibit specific siRNA synthesis. It was reported that DCL1, DCL4, AGO7, HYL1, HEN1, Hasty1 (HST1), RDR6, and Pol IV, are involved in the biogenesis of long siRNAs (lsiRNA-1), which is also induced by effector *avrRpt2* carrying bacterial infection. In *Arabidopsis*, AtlsiRNA-1 induction targets RNA binding domain abundant in apicomplexans (*AtRAP*) mRNA and promotes down-regulation of the targeted mRNA (Figure 4). This down-regulation of *AtRAP* enhances resistance to both virulent and avirulent strains of bacteria as observed in *AtRAP* knockout mutant. The AtlsiRNA-1 down-regulates the target mRNA via decapping and XRN4-mediated degradation [250].

Another type-III bacterial effectors (*HopT1-1*) interacts with AGO1 through conserved Glycine/Tryptophane (GW) motif and suppresses the AGO1-dependent miRNA pathway to promote pathogenicity in *Arabidopsis*. AGO1-dependent miRNA positively regulates PAMPs-induced callose deposition and bacterial resistance. The bacterial flagellin-derived peptide flg22 differentially expresses certain AGO1 dependent miRNAs, and the overexpression of the selected miRNAs revealed that miR160a positively while miR398b and miR773 negatively regulate callose deposition and disease resistance to *P. syringae* [251]. The flg22 also triggers the AGO1 binding to promote transcription of innate immune and chitin responsive genes [252]. The derived-peptide induced miRNAs (miR393) down-regulate mRNA for F-box auxin receptors and suppress auxin signaling to enhance basal defense against bacteria [253]. The miR393 pair was identified to function in antibacterial immunity through two distinct AGOs proteins. AGO1 associated miR393 and AGO2 associated miR393* contribute to PTI and ETI, respectively [254]. During plant growth and development, the miR390 participates in auxin signaling through the production of *trans*-acting siRNAs (tasiRNAs) from *T*rans acting siRNA *3* (*TAS3*) transcripts to regulate auxin responsive factor (*ARF*) genes by DCL1 processing and AGO7 as an associated partner [255]. It is unclear how the two contrasting, miR393 and miR390-*TAS3*-*ARF* pathways of auxin signaling correlate with each other during bacterial infection. However, a small class of Auxin response factors (ARF) also involves in repression of downstream auxin-regulated genes [256], and it might be possible that miR393 activate these factors to inhibit miR390-*TAS3*-*ARF* pathway. AGO4 is the important component of RdDM pathway that works independently in regulating resistance to *P. syringae* pv. * tomato* DC3000. Loss of function in RdDM pathway components, upstream or downstream of AGO4 does not facilitate resistance to *P. syringae* [257]. Therefore, after bacterial infection, flg22 down-regulates AGO4 to repress RdDM pathway and inhibit TGS [258].

RDR6 is a key RNA silencing factor studied in plants against bacterial pathogens. RDR6 is involved in the biogenesis of bacterial-induced lsiRNA-1 and natural antisense transcript (NAT)-associated siRNAs (nat-siRNA, e.g., nat-siRNAATGB2), and knockout *rdr6* mutants are highly susceptible to *P. syringae* pv. *tomato*, *A. tumefaciens,* and *Xanthomonas oryzae* pv. *oryzae* [249,250,259,260]. Studies on various plant species showed that several miRNAs target ETI associates with NB-LRR encoded *R* genes and generate secondary siRNAs or phased siRNAs (phasiRNA) in an RDR6-dependent manner [261,262,263,264]. Three highly abundant 22 nt miRNA families were identified in *Medicago* that target at least 114 conserved domains of defense-related NB-LRR encoded *R* genes to enhance production of secondary siRNAs or phasiRNAs [261]. *Arabidopsis* miR825^*^ and tomato miR482 target the NB-LRR type *R* genes and their expression negatively correlate with target genes. Upon bacterial infection, these miRNAs are suppressed to activate the target defense-related genes [263,265]. In *Arabidopsis*, *RDR6,* and miR472 suppress both basal and RPS5-mediated resistance to *Pto* DC3000 through controlling coiled-coil nucleotide-binding leucine-rich-repeats (*CNLs*) family genes [266]. Comparing the role of RNAi mechanisms against different pathogens, it is observed that lack of function of one protein can be substituted by other during viral and fungal infections, while, in case of bacterial infection, there is a lack of information about the effect of down-regulation or suppression of the particular RNAi component on the function and expression of other members of this pathway. Table 2 describes the basic functions of RNAi pathway key components involved in the counter defense of plant pathogens.

## 6. Conclusions

In recent years, highly advanced technologies and molecular works have unfolded many pathogen-induced plant defense responses and strategies during stress conditions. However, the interaction of plants with various virulent pathogens is so complicated that the exact mechanism of plant natural immunity or resistance remains elusive. Although researchers have identified a large number of resistance genes in different crops, the question still remains of how to classify the role of specific genes in a particular plant–pathogen interaction. In this review, we summarized the RNAi mechanism, a common plant endogenous defense machinery, against all kind of pathogens. We discussed the important components and function of each protein member in silencing of invading nucleic acid based on previously published reports. The three main components of RNAi machinery and their cofactors collaborate with each other against the foreign RNA/DNA or suppressors of the invading pathogens. This mechanism also holds certain limitation regarding their activation and function. Pathogen-induced differential expression of some components results in the contradictory immunity responses raises questions on silencing pathways. Second, the function of many DCLs, AGOs, and RDRs are still largely unknown, particularly the antibacterial activities of RDR6-dependent miRNA target defense genes. One of the important points highlighted in this review is that RDRγ protein function is still not fully studied and this group needs more research to fully understand the complete mechanism.

In conclusion, the plant RNAi mechanism provides basic information to develop naturally immune crops against the virulent pathogens. Further research needs to understand the exact mechanism and cooperative strategies of RNAi pathway components that will hopefully resolve the complex plant defense system against various pathogens.

## Figures and Tables

**Table 1:** Number of Dicer-Like protiens (DCLs), Argonaute (AGOs) and RNA-dependent RNA polymerase (RDRs) identified in various plant species.

Plant Species	DCLs	AGOs	RDRs	References
*Arabidopsis thaliana*	4	10	6	[11]
*Brassica napus*	13	28	16	[68,69]
*Capsicum annuum*	4	12	6	[70]
*Coffea canephora*	9	11	8	[71]
*Cucumis sativus*	5	7	8	[72]
*Glycine max*	7	21	7	[73]
*Nicotiana benthamiana*	4	9	3	[74]
*Oryza sativa*	8	19	5	[75]
*Phaseolus vulgaris*	6	17		[76]
*Salvia miltiorrhiza*	5	10	5	[77,78,79]
*Setaria italica*	8	19	11	[80]
*Solanum lycopersicum*	7	25	6	[55]
*Solanum tuberosum*	7	11	7	[81,82]
*Sorghum bicolor*	5	14	7	[73]
*Vitis vinifera*	4	13	5	[83]
*Zea mays*	5	18	5	[54]

**Table 2:** Diversity of RNA interference (RNAi) pathway essential components involved in plant immunity.

Proteins	Components	Functions	References
Dicer-like protein	DCL1	Biogenesis of 21 nt siRNAs, miRNAs, nat-siRNA and lsiRNA, involved in PAMP-triggered immunity	[69,216,251,253,267]
	DCL2	Production of 22 nt siRNAs and stimulates transitivity	[84,167,268]
	DCL3	Biogenesis of 24 nt siRNA and hc-siRNA, involved in chromatin modification and transcriptional silencing	[29,30,88]
	DCL4	Biogenesis of 21 nt siRNAs and processed ta-siRNAs	[28,69,89,269]
Argonaute protein	AGO1	Major AGO protein that associates with vsiRNAs, involved in miRNA-directed gene silencing and posttranscriptional gene silencing	[35,116,251,270]
	AGO2	miRNA-directed gene silencing, repress translation, and played role in immune responses	[84,92,254,271]
	AGO4	Bind 24 nt siRNAs to form RdDM complex, involved in DNA methylation and transcriptional gene silencing	[129,135,257,272]
	AGO5	Bind 21-, 22-, and 24 nt siRNAs, involved in posttranscriptional gene silencing and systemic resistance	[119,273]
	AGO7	Required for generation of lsiRNAs and contributed to effector-triggered immunity	[250,251]
RNA-dependent RNA polymerase	RDR1	Amplification of siRNA and production of dsRNA, limit systemic infection	[69,89,136,152]
	RDR2	Production of secondary dsRNA through RdDM pathway and involved in regulation of transposons	[61,62,89]
	RDR6	Biogenesis of ta-siRNAs, nat-siRNAs, and secondary siRNA, involved in cell to cell silencing signal and posttranscriptional gene silencing	[250,259,274]
Double-stranded RNA binding proteins	DRB1 (HYL1)DRB2-DRB4	Interact with DCls for the efficient production miRNA, tasiRNAs, siRNA, nat-siRNA and lsiRNA	[102,103,104,106]
HUA enhancer 1	HEN1	Biogenesis of lsiRNA and nat-siRNA, stabilized and methylates all endogenous sRNAs	[97,259,275]
Heat shock protein	HSP70-90	Role in RISC formation and siRNA loading	[42,43,276]
Cofactors	SGS	Stabilized the RISC-cleavage and facilitated RDR activity	[50,277]
	SDE	Accumulation of tasiRNAs and facilitated RDR activity in conversion of ssRNAs in to dsRNA	[154,155]

Small interfering RNAs (siRNAs), microRNAs (miRNAs), Natural antisense transcript-derived siRNAs (nat-siRNAs), Heterochromatic siRNAs (hcsiRNAs), Virus-derived siRNAs (vsiRNAs), Long siRNAs (lsiRNAs), Double-stranded RNAs (dsRNA), Small RNAs (sRNAs), Single-stranded RNAs (ssRNAs), *Trans*-acting siRNAs (tasiRNAs), RNA-directed DNA methylation (RdDM), Suppressor of gene silencing (SGS), Silencing defectives (SDE), RNA-induced silencing complexes (RISCs).

## References

[1] Napoli C., Lemieux C., Jorgensen R. (1990). Introduction of a chimeric chalcone synthase gene into petunia results in reversible co-suppression of homologous genes in trans. Plant Cell.

[2] Romano N., Macino G. (1992). Quelling: Transient inactivation of gene expression in *Neurospora crassa* by transformation with homologous sequences. Mol. Microbiol..

[3] Guo S., Kemphues K.J. (1995). par-1, a gene required for establishing polarity in *C. elegans* embryos, encodes a putative Ser/Thr kinase that is asymmetrically distributed. Cell.

[4] Fire A. (1998). Potent and specific genetic interference by double stranded RNA in *Caenorhabditis elegans*. Nature.

[5] Hammond S.M., Bernstein E., Beach D., Hannon G.J. (2000). An RNA-directed nuclease mediates post-transcriptional gene silencing in Drosophila cells. Nature.

[6] Zamore P.D., Tuschl T., Sharp P.A., Bartel D.P. (2000). RNAi: Double-stranded RNA directs the ATP-dependent cleavage of mRNA at 21 to 23 nucleotide intervals. Cell.

[7] Bernstein E., Caudy A.A., Hammond S.M., Hannon G.J. (2001). Role for bidentate ribnuclease in the initiation site of RNA interference. Nature.

[8] Baulcombe D. (2004). RNA silencing in plants. Nature.

[9] Chapman E.J., Carrington J.C. (2007). Specialization and evolution of endogenous small RNA pathways. Nat. Rev. Genet..

[10] Vaucheret H. (2006). Post-transcriptional small RNA pathways in plants: Mechanisms and regulations. Genes Dev..

[11] Vaucheret H. (2008). Plant Argonautes. Trends Plant Sci..

[12] Voinnet O. (2009). Origin, biogenesis, and activity of plant microRNAs. Cell.

[13] Wassenegger M., Krczal G. (2006). Nomenclature and functions of RNA-directed RNA polymerases. Trends Plant Sci..

[14] Carmell M.A., Hannon G.J. (2004). RNase III enzymes and the initiation of gene silencing. Nat. Struct. Mol. Biol..

[15] Margis R., Fusaro A.F., Smith N.A., Curtin S.J., Watson J.M., Finnegan E.J., Waterhouse P.M. (2006). The evolution and diversification of Dicers in plants. FEBS Lett..

[16] Cordin O., Banroques J., Tanner N.K., Linder P. (2006). The DEAD-box protein family of RNA helicases. Gene.

[17] Montavon T., Kwon Y., Zimmermann A., Hammann P., Vincent T., Cognat V., Bergdoll M., Michel F., Dunoyer P. (2018). Characterization of DCL4 missense alleles provides insights into its ability to process distinct classes of dsRNA substrates. Plant J..

[18] Park J.E., Heo I., Tian Y., Simanshu D.K., Chang H., Jee D., Patel D.J., Kim V.N. (2011). Dicer recognizes the 5′ end of RNA for efficient and accurate processing. Nature.

[19] Tian Y., Simanshu D.K., Ma J.B., Park J.E., Heo I., Kim V.N., Patel D.J. (2014). A phosphate-binding pocket within the platform-PAZ-connector helix cassette of human Dicer. Mol. Cell.

[20] Kandasamy S.K., Fukunaga R. (2016). Phosphate-binding pocket in Dicer-2 PAZ domain for high-fidelity siRNA production. Proc. Natl. Acad. Sci. USA.

[21] Kurzynska-Kokorniak A., Pokornowska M., Koralewska N., Hoffmann W., Bienkowska-Szewczyk K., Figlerowicz M. (2016). Revealing a new activity of the human Dicer DUF283 domain in vitro. Sci. Rep..

[22] Aguado L.C., TenOever B.R. (2018). RNase III nucleases and the evolution of antiviral systems. BioEssays.

[23] Nicholson A.W. (2014). Ribonuclease III mechanisms of double-stranded RNA cleavage. Wiley Interdiscip. Rev. RNA.

[24] Barraud P., Banerjee S., Mohamed W.I., Jantsch M.F., Allain F.H.-T. (2014). A bimodular nuclear localization signal assembled via an extended double-stranded RNA-binding domain acts as an RNA-sensing signal for transportin 1. Proc. Natl. Acad. Sci. USA.

[25] Banerjee S., Barraud P. (2014). Functions of double-stranded RNA-binding domains in nucleocytoplasmic transport. RNA Biol..

[26] Hohn T., Vazquez F. (2011). RNA silencing pathways of plants: Silencing and its suppression by plant DNA viruses. Biochim. Biophys. Acta.

[27] Mlotshwa S., Schauer S.E., Smith T.H., Mallory A.C., Herr J.M., Roth B., Merchant D.S., Ray A., Bowman L.H., Vance V.B. (2005). Ectopic Dicer-like1 expression in P1/HC-Pro *Arabidopsis* rescues phenotypic anomalies but not defects in microRNA and silencing pathways. Plant Cell Online.

[28] Qi Y., Denli A.M., Hannon G.J. (2005). Biochemical specialization within *Arabidopsis* RNA silencing pathways. Mol. Cell.

[29] Xie Z., Johansen L.K., Gustafson A.M., Kasschau K.D., Lellis A.D., Zilberman D., Jacobsen S.E., Carrington J.C. (2004). Genetic and functional diversification of small RNA pathways in plants. PLoS Biol..

[30] Henderson I.R., Zhang X., Lu C., Johnson L., Meyers B.C., Green P.J., Jacobsen S.E. (2006). Dissecting *Arabidopsis thaliana* Dicer function in small RNA processing, gene silencing and DNA methylation patterning. Nat. Genet..

[31] Coursey T., Regedanz E., Bisaro D.M. (2018). *Arabidopsis* RNA polymerase V mediates enhanced compaction and silencing of geminivirus and transposon chromatin during host recovery from infection. J. Virol..

[32] Vazquez F., Vaucheret H., Rajagopalan R., Lepers C., Gasciolli V., Mallory A.C., Hilbert J.L., Bartel D.P., Crété P. (2004). Endogenous trans-acting siRNAs regulate the accumulation of *Arabidopsis* mRNAs. Mol. Cell.

[33] Dunoyer P., Himber C., Voinnet O. (2005). Dicer-like 4 is required for RNA interference and produces the 21-nucleotide small interfering RNA component of the plant cell-to-cell silencing signal. Nat. Genet..

[34] Gasciolli V., Mallory A.C., Bartel D.P., Vaucheret H. (2005). Partially redundant functions of *Arabidopsis* Dicer-like enzymes and a role for DCL4 in producing trans-acting siRNAs. Curr. Biol..

[35] Mallory A., Vaucheret H. (2010). Form, function, and regulation of Argonaute proteins. Plant Cell.

[36] Parker J.S. (2010). How to slice: Snapshots of Argonaute in action. Silence.

[37] Simon B., Kirkpatrick J.P., Eckhardt S., Reuter M., Rocha E.A., Andrade-Navarro M.A., Sehr P., Pillai R.S., Carlomagno T. (2011). Recognition of 2′-o-methylated 3′-end of piRNA by the PAZ domain of a Piwi protein. Structure.

[38] Zilberman D., Cao X., Jacobsen S.E. (2003). Argonaute4 control of locus-specific siRNA accumulation and DNA and histone methylation. Science.

[39] Havecker E.R., Wallbridge L.M., Hardcastle T.J., Bush M.S., Kelly K.A., Dunn R.M., Schwach F., Doonan J.H., Baulcombe D.C. (2010). The *Arabidopsis* RNA-directed DNA methylation Argonautes functionally diverge based on their expression and interaction with target loci. Plant Cell.

[40] Fagard M., Boutet S., Morel J.B., Bellini C., Vaucheret H. (2000). AGO1, QDE-2, and RDE-1 are related proteins required for post-transcriptional gene silencing in plants, quelling in fungi, and RNA interference in animals. Proc. Natl. Acad. Sci. USA.

[41] Hunter C., Sun H., Poethig R.S. (2003). The *Arabidopsis* heterochronic gene ZIPPY is an Argonaute family member. Curr. Biol..

[42] Röhl A., Rohrberg J., Buchner J. (2013). The chaperone Hsp90: Changing partners for demanding clients. Trends Biochem. Sci..

[43] Ye R., Wang W., Iki T., Liu C., Wu Y., Ishikawa M., Zhou X., Qi Y. (2012). Cytoplasmic assembly and selective nuclear import of *Arabidopsis* Argonaute4/siRNA complexes. Mol. Cell.

[44] Khvorova A., Reynolds A., Jayasena S.D. (2003). Functional siRNAs and miRNAs exhibit strand bias. Cell.

[45] Schwarz D.S., Hutvágner G., Du T., Xu Z., Aronin N., Zamore P.D. (2003). Asymmetry in the assembly of the RNAi enzyme complex. Cell.

[46] Liu W., Duttke S.H., Hetzel J., Groth M., Feng S., Gallego-Bartolome J., Zhong Z., Kuo H.Y., Wang Z., Zhai J. (2018). RNA-directed DNA methylation involves co-transcriptional small-RNA-guided slicing of polymerase V transcripts in *Arabidopsis*. Nat. Plants.

[47] Astier-Manifacier S., Cornuet P. (1971). RNA-dependent RNA polymerase in Chinese cabbage. Biochim. Biophys. Acta Nucleic Acids Protein Synth..

[48] Schiebel W., Pélissier T., Riedel L., Thalmeir S., Schiebel R., Kempe D., Lottspeich F., Sänger H.L., Wassenegger M. (1998). Isolation of an RNA-directed RNA polymerase-specific cDNA clone from tomato. Plant Cell.

[49] Dalmay T., Hamilton A., Rudd S., Angell S., Baulcombe D.C. (2000). An RNA-dependent RNA polymerase gene in *Arabidopsis* is required for posttranscriptional gene silencing mediated by a transgene but not by a virus. Cell.

[50] Mourrain P., Béclin C., Elmayan T., Feuerbach F., Godon C., Morel J.B., Jouette D., Lacombe A.M., Nikic S., Picault N. (2000). *Arabidopsis* SGS2 and SGS3 genes are required for posttranscriptional gene silencing and natural virus resistance. Cell.

[51] Wang M.B., Metzlaff M. (2005). RNA silencing and antiviral defense in plants. Curr. Opin. Plant Biol..

[52] Zong J., Yao X., Yin J., Zhang D., Ma H. (2009). Evolution of the RNA-dependent RNA polymerase (RdRP) genes: Duplications and possible losses before and after the divergence of major eukaryotic groups. Gene.

[53] Venkataraman S., Prasad B., Selvarajan R. (2018). RNA dependent RNA polymerases: Insights from structure, function and evolution. Viruses.

[54] Qian Y., Cheng Y., Cheng X., Jiang H., Zhu S., Cheng B. (2011). Identification and characterization of Dicer-like, Argonaute and RNA-dependent RNA polymerase gene families in maize. Plant Cell Rep..

[55] Bai M., Yang G.S., Chen W.T., Mao Z.C., Kang H.X., Chen G.H., Yang Y.H., Xie B.Y. (2012). Genome-wide identification of Dicer-like, Argonaute and RNA-dependent RNA polymerase gene families and their expression analyses in response to viral infection and abiotic stresses in *Solanum lycopersicum*. Gene.

[56] Pandey S.P., Baldwin I.T. (2007). RNA-directed RNA polymerase 1 (RdR1) mediates the resistance of *Nicotiana attenuata* to herbivore attack in nature. Plant J..

[57] Liu Y., Gao Q., Wu B., Ai T., Guo X. (2009). *NgRDR1*, an RNA-dependent RNA polymerase isolated from *Nicotiana glutinosa*, was involved in biotic and abiotic stresses. Plant Physiol. Biochem..

[58] Hunter L.J.R., Westwood J.H., Heath G., Macaulay K., Smith A.G., MacFarlane S.A., Palukaitis P., Carr J.P. (2013). Regulation of RNA-dependent RNA polymerase 1 and isochorismate synthase gene expression in *Arabidopsis*. PLoS ONE.

[59] Lu C., Kulkarni K., Muthuvalliappan R., Tej S.S., Poethig R.S., Henderson I.R., Jacobsen S.E., Wang W., Green P.J., Meyers B.C. (2006). MicroRNAs and other small RNAs enriched in the *Arabidopsis* RNA-dependent RNA polymerase-2 mutant. Genome Res..

[60] Deng S., Xu J., Liu J., Kim S.H., Shi S., Chua N.H. (2015). JMJ24 binds to RDR2 and is required for the basal level transcription of silenced loci in *Arabidopsis*. Plant J..

[61] Jia Y., Lisch D.R., Ohtsu K., Scanlon M.J., Nettleton D., Schnable P.S. (2009). Loss of RNA-dependent RNA polymerase 2 (RDR2) function causes widespread and unexpected changes in the expression of transposons, genes, and 24-nt small RNAs. PLoS Genet..

[62] Jauvion V., Rivard M., Bouteiller N., Elmayan T., Vaucheret H. (2012). RDR2 partially antagonizes the production of RDR6-dependent siRNA in sense transgene-mediated PTGS. PLoS ONE.

[63] Nuthikattu S., McCue A.D., Panda K., Fultz D., DeFraia C., Thomas E.N., Slotkin R.K. (2013). The initiation of epigenetic silencing of active transposable elements is triggered by RDR6 and 21–22 nucleotide small interfering RNAs. Plant Physiol..

[64] Brosnan C.A., Mitter N., Christie M., Smith N.A., Waterhouse P.M., Carroll B.J. (2007). Nuclear gene silencing directs reception of long-distance mRNA silencing in *Arabidopsis*. Proc. Natl. Acad. Sci. USA.

[65] Liu Q., Yao X., Pi L., Wang H., Cui X., Huang H. (2009). The Argonaute 10 gene modulates shoot apical meristem maintenance and establishment of leaf polarity by repressing miR165/166 in *Arabidopsis*. Plant J..

[66] Dalakouras A., Lauter A., Bassler A., Krczal G., Wassenegger M. (2018). Transient expression of intron-containing transgenes generates non-spliced aberrant pre-mRNAs that are processed into siRNAs. Planta.

[67] Polydore S., Axtell M.J. (2018). Analysis of RDR1/RDR2/RDR6-independent small RNAs in *Arabidopsis thaliana* improves MIRNA annotations and reveals unexplained types of short interfering RNA loci. Plant J..

[68] Zhao X., Zheng W., Zhong Z., Chen X., Wang A., Wang Z. (2016). Genome-wide analysis of RNA-interference pathway in *Brassica napus*, and the expression profile of *BnAGOs* in response to *Sclerotinia sclerotiorum* infection. Eur. J. Plant Pathol..

[69] Cao J.-Y., Xu Y.-P., Li W., Li S.-S., Rahman H., Cai X.-Z. (2016). Genome-wide identification of Dicer-like, Argonaute, and RNA-dependent RNA polymerase gene families in *Brassica species* and functional analyses of their *Arabidopsis* homologs in resistance to *Sclerotinia sclerotiorum*. Front. Plant Sci..

[70] Qin L., Mo N., Muhammad T., Liang Y. (2018). Genome-wide analysis of DCL, AGO, and RDR gene families in pepper (*Capsicum Annuum* L.). Int. J. Mol. Sci..

[71] Fernandes-Brum C.N., Rezende P.M., Ribeiro T.H.C., De Oliveira R.R., De Sousa Cardoso T.C., Do Amaral L.R., De Souza Gomes M., Chalfun A. (2017). A genome-wide analysis of the RNA-guided silencing pathway in coffee reveals insights into its regulatory mechanisms. PLoS ONE.

[72] Gan D., Zhan M., Yang F., Zhang Q., Hu K., Xu W., Lu Q., Zhang L., Liang D. (2017). Expression analysis of argonaute, Dicer-like, and RNA-dependent RNA polymerase genes in cucumber (*Cucumis sativus* L.) in response to abiotic stress. J. Genet..

[73] Liu X., Lu T., Dou Y., Yu B., Zhang C. (2014). Identification of RNA silencing components in soybean and sorghum. BMC Bioinform..

[74] Nakasugi K., Crowhurst R.N., Bally J., Wood C.C., Hellens R.P., Waterhouse P.M. (2013). De novo transcriptome sequence assembly and analysis of RNA silencing genes of *Nicotiana benthamiana*. PLoS ONE.

[75] Kapoor M., Arora R., Lama T., Nijhawan A., Khurana J.P., Tyagi A.K., Kapoor S. (2008). Genome-wide identification, organization and phylogenetic analysis of Dicer-like, Argonaute and RNA-dependent RNA Polymerase gene families and their expression analysis during reproductive development and stress in rice. BMC Genom..

[76] De Sousa Cardoso T.C., Portilho L.G., de Oliveira C.L., Mckeown P.C., Maluf W.R., Gomes L.A.A., Teixeira T.A., do Amaral L.R., Spillane C., de Souza Gomes M. (2016). Genome-wide identification and in silico characterisation of microRNAs, their targets and processing pathway genes in *Phaseolus vulgaris* L.. Plant Biol..

[77] Shao F., Lu S. (2013). Genome-wide identification, molecular cloning, expression profiling and posttranscriptional regulation analysis of the Argonaute gene family in *Salvia miltiorrhiza*, an emerging model medicinal plant. BMC Genom..

[78] Shao F., Lu S. (2014). Identification, molecular cloning and expression analysis of five RNA-dependent RNA polymerase genes in *Salvia miltiorrhiza*. PLoS ONE.

[79] Shao F., Qiu D., Lu S. (2015). Comparative analysis of the Dicer-like gene family reveals loss of miR162 target site in *SmDCL1* from *Salvia miltiorrhiza*. Sci. Rep..

[80] Yadav C.B., Muthamilarasan M., Pandey G., Prasad M. (2015). Identification, characterization and expression profiling of Dicer-like, Argonaute and RNA-dependent RNA polymerase gene families in foxtail millet. Plant Mol. Biol. Report..

[81] Mirzaei K., Bahramnejad B., Shamsifard M.H., Zamani W. (2014). In silico identification, phylogenetic and bioinformatic analysis of argonaute genes in plants. Int. J. Genom..

[82] Esposito S., Aversano R., D’Amelia V., Villano C., Alioto D., Mirouze M., Carputo D. (2018). Dicer-like and RNA-dependent RNA polymerase gene family identification and annotation in the cultivated *Solanum tuberosum* and its wild relative *S. commersonii*. Planta.

[83] Zhao H., Zhao K., Wang J., Chen X., Chen Z., Cai R., Xiang Y. (2015). Comprehensive analysis of Dicer-like, Argonaute, and RNA-dependent RNA polymerase gene families in grapevine (*Vitis Vinifera*). J. Plant Growth Regul..

[84] Wang X.-B., Jovel J., Udomporn P., Wang Y., Wu Q., Li W.-X., Gasciolli V., Vaucheret H., Ding S.-W. (2011). The 21-nucleotide, but not 22-nucleotide, viral secondary small interfering RNAs direct potent antiviral defense by two cooperative Argonautes in *Arabidopsis thaliana*. Plant Cell.

[85] Bisaro D.M. (2006). Silencing suppression by geminivirus proteins. Virology.

[86] Akbergenov R., Si-Ammour A., Blevins T., Amin I., Kutter C., Vanderschuren H., Zhang P., Gruissem W., Meins F., Hohn T. (2006). Molecular characterization of geminivirus-derived small RNAs in different plant species. Nucleic Acids Res..

[87] Bouché N., Lauressergues D., Gasciolli V., Vaucheret H. (2006). An antagonistic function for *Arabidopsis* DCL2 in development and a new function for DCL4 in generating viral siRNAs. EMBO J..

[88] Diaz-Pendon J.A., Li F., Li W.-X., Ding S.-W. (2007). Suppression of antiviral silencing by cucumber mosaic virus 2b protein in *Arabidopsis* is associated with drastically reduced accumulation of three classes of viral small interfering RNAs. Plant Cell.

[89] Garcia-Ruiz H., Takeda A., Chapman E.J., Sullivan C.M., Fahlgren N., Brempelis K.J., Carrington J.C. (2010). *Arabidopsis* RNA-dependent RNA polymerases and Dicer-like proteins in antiviral defense and small interfering RNA biogenesis during turnip mosaic virus infection. Plant Cell.

[90] Dzianott A., Sztuba-Solińska J., Bujarski J.J. (2012). Mutations in the antiviral RNAi defense pathway modify Brome mosaic virus RNA recombinant profiles. Mol. Plant-Microbe Interact..

[91] Andika I.B., Maruyama K., Sun L., Kondo H., Tamada T., Suzuki N. (2015). Different Dicer-like protein components required for intracellular and systemic antiviral silencing in *Arabidopsis thaliana*. Plant Signal. Behav..

[92] Zhang X., Zhang X., Singh J., Li D., Qu F. (2012). Temperature-dependent survival of turnip crinkle virus-infected *Arabidopsis* plants relies on an RNA silencing-based defense that requires DCL2, AGO2, and HEN1. J. Virol..

[93] Qin C., Li B., Fan Y., Zhang X., Yu Z., Ryabov E., Zhao M., Wang H., Shi N., Zhang P. (2017). Roles of Dicer-like proteins 2 and 4 in intra- and intercellular antiviral silencing. Plant Physiol..

[94] Qiao W., Zarzyńska-Nowak A., Nerva L., Kuo Y.-W., Falk B.W. (2018). Accumulation of 24 nucleotide transgene-derived siRNAs is associated with Crinivirus immunity in transgenic plants. Mol. Plant Pathol..

[95] Blevins T., Rajeswaran R., Shivaprasad P.V., Beknazariants D., Si-Ammour A., Park H.S., Vazquez F., Robertson D., Meins F., Hohn T. (2006). Four plant Dicers mediate viral small RNA biogenesis and DNA virus induced silencing. Nucleic Acids Res..

[96] Aregger M., Borah B.K., Seguin J., Rajeswaran R., Gubaeva E.G., Zvereva A.S., Windels D., Vazquez F., Blevins T., Farinelli L. (2012). Primary and secondary siRNAs in Geminivirus-induced gene silencing. PLoS Pathog..

[97] Li J., Yang Z., Yu B., Liu J., Chen X. (2005). Methylation protects miRNAs and siRNAs from a 3′-end uridylation activity in *Arabidopsis*. Curr. Biol..

[98] Boutet S., Vazquez F., Jun L., Béclin C., Fagard M., Gratias A., Morel J.-B., Crété P., Chen X., Vaucheret H. (2003). *Arabidopsis* HEN1: A genetic link between endogenous miRNA controlling development and siRNA controlling transgene silencing and virus resistance. Curr. Biol..

[99] Blevins T., Rajeswaran R., Aregger M., Borah B.K., Schepetilnikov M., Baerlocher L., Farinelli L., Meins F., Hohn T., Pooggin M.M. (2011). Massive production of small RNAs from a non-coding region of cauliflower mosaic virus in plant defense and viral counter-defense. Nucleic Acids Res..

[100] Li Y., Muhammad T., Wang Y., Zhang D., Crabbe M.J.C., Liang Y. (2018). Salicylic acid collaborates with gene silencing to tomato defense against tomato yellow leaf curl virus (TYLCV). Pak. J. Bot..

[101] Eamens A.L., Kim K.W., Curtin S.J., Waterhouse P.M. (2012). DRB2 is required for microRNA biogenesis in *Arabidopsis thaliana*. PLoS ONE.

[102] Eamens A.L., Wook Kim K., Waterhouse P.M. (2012). DRB2, DRB3 and DRB5 function in a non-canonical microRNA pathway in *Arabidopsis thaliana*. Plant Signal. Behav..

[103] Raja P., Jackel J.N., Li S., Heard I.M., Bisaro D.M. (2014). *Arabidopsis* double-stranded RNA binding protein DRB3 participates in methylation-mediated defense against Geminiviruses. J. Virol..

[104] Eamens A.L., Smith N.A., Curtin S.J., Wang M., Waterhouse P.M. (2009). The *Arabidopsis thaliana* double-stranded RNA binding protein DRB1 directs guide strand selection from microRNA duplexes. RNA.

[105] Reis R.S., Hart-Smith G., Eamens A.L., Wilkins M.R., Waterhouse P.M. (2015). Gene regulation by translational inhibition is determined by Dicer partnering proteins. Nat. Plants.

[106] Pélissier T., Clavel M., Chaparro C., Pouch-PéLissier M.N., Vaucheret H., Deragon J.M. (2011). Double-stranded RNA binding proteins DRB2 and DRB4 have an antagonistic impact on polymerase IV-dependent siRNA levels in *Arabidopsis*. RNA.

[107] Clavel M., Pélissier T., Descombin J., Jean V., Picart C., Charbonel C., Saez-Vásquez J., Bousquet-Antonelli C., Deragon J.M. (2015). Parallel action of *AtDRB2* and RdDM in the control of transposable element expression. BMC Plant Biol..

[108] Jakubiec A., Yang S.W., Chua N.H. (2012). *Arabidopsis* DRB4 protein in antiviral defense against Turnip yellow mosaic virus infection. Plant J..

[109] Zhang X., Zhang X., Wu K., Liu Z., Li D., Qu F. (2016). Incomplete DRB4-dependence of the DCL4-mediated antiviral defense. Sci. Rep..

[110] Tschopp M.A., Iki T., Brosnan C.A., Jullien P.E., Pumplin N. (2017). A complex of *Arabidopsis* DRB proteins can impair dsRNA processing. RNA.

[111] Clavel M., Pélissier T., Montavon T., Tschopp M.A., Pouch-Pélissier M.N., Descombin J., Jean V., Dunoyer P., Bousquet-Antonelli C., Deragon J.M. (2016). Evolutionary history of double-stranded RNA binding proteins in plants: Identification of new cofactors involved in easiRNA biogenesis. Plant Mol. Biol..

[112] Zhang S., Dou Y., Li S., Ren G., Chevalier D., Zhang C., Yu B. (2018). DAWDLE interacts with Dicer-like proteins to mediate small RNA biogenesis. Plant Physiol..

[113] Morris E.R., Chevalier D., Walker J.C. (2006). DAWDLE, a forkhead-associated domain gene, regulates multiple aspects of plant development. Plant Physiol..

[114] Feng B., Ma S., Chen S., Zhu N., Zhang S., Yu B., Yu Y., Le B., Chen X., Dinesh-Kumar S.P. (2016). PARylation of the forkhead-associated domain protein DAWDLE regulates plant immunity. EMBO Rep..

[115] Jaubert M., Bhattacharjee S., Mello A.F.S., Perry K.L., Moffett P. (2011). Argonaute 2 mediates RNA-silencing antiviral defenses against potato virus X in *Arabidopsis*. Plant Physiol..

[116] Garcia-Ruiz H., Carbonell A., Hoyer J.S., Fahlgren N., Gilbert K.B., Takeda A., Giampetruzzi A., Garcia Ruiz M.T., McGinn M.G., Lowery N. (2015). Roles and programming of *Arabidopsis* Argonaute proteins during turnip mosaic virus infection. PLoS Pathog..

[117] Harvey J.J.W., Lewsey M.G., Patel K., Westwood J., Heimstädt S., Carr J.P., Baulcombe D.C. (2011). An antiviral defense role of AGO2 in plants. PLoS ONE.

[118] Takeda A., Iwasaki S., Watanabe T., Utsumi M., Watanabe Y. (2008). The mechanism selecting the guide strand from small RNA duplexes is different among Argonaute proteins. Plant Cell Physiol..

[119] Brosseau C., Moffett P. (2015). Functional and genetic analysis identify a role for *Arabidopsis* Argonaute 5 in antiviral RNA silencing. Plant Cell.

[120] Qu F., Ye X., Morris T.J. (2008). *Arabidopsis* DRB4, AGO1, AGO7, and RDR6 participate in a DCL4-initiated antiviral RNA silencing pathway negatively regulated by DCL1. Proc. Natl. Acad. Sci. USA.

[121] Morel J., Godon C., Mourrain P., Béclin C., Boutet S., Feuerbach F., Proux F., Vaucheret H. (2002). Fertile hypomorphic Argonaute (*ago1*) mutants impaired in post-transcriptional gene silencing and virus resistance. Plant Cell.

[122] Lewsey M.G., Murphy A.M., Maclean D., Dalchau N., Westwood J.H., Macaulay K., Bennett M.H., Moulin M., Hanke D.E., Powell G. (2010). Disruption of two defensive signaling pathways by a viral RNA silencing suppressor. Mol. Plant-Microbe Interact..

[123] Ludman M., Burgyán J., Fátyol K. (2017). Crispr/Cas9 mediated inactivation of Argonaute 2 reveals its differential involvement in antiviral responses. Sci. Rep..

[124] Paudel D.B., Ghoshal B., Jossey S., Ludman M., Fatyol K., Sanfaçon H. (2018). Expression and antiviral function of Argonaute 2 in *Nicotiana benthamiana* plants infected with two isolates of tomato ringspot virus with varying degrees of virulence. Virology.

[125] Jones L., Keining T., Eamens A., Vaistij F.E. (2006). Virus-induced gene silencing of Argonaute genes in *Nicotiana benthamiana* demonstrates that extensive systemic silencing requires Argonaute 1-like and Argonaute 4-like genes. Plant Physiol..

[126] Au P.C.K., Dennis E.S., Wang M.B. (2017). Analysis of argonaute 4-associated long non-coding RNA in *Arabidopsis thaliana* sheds novel insights into gene regulation through RNA-directed DNA methylation. Genes.

[127] Mi S., Cai T., Hu Y., Chen Y., Hodges E., Ni F., Wu L., Li S., Zhou H., Long C. (2008). Sorting of small RNAs into *Arabidopsis* Argonaute complexes is directed by the 5′ terminal Nucleotide. Cell.

[128] Wang H., Zhang X., Liu J., Kiba T., Woo J., Ojo T., Hafner M., Tuschl T., Chua N.H., Wang X.J. (2011). Deep sequencing of small RNAs specifically associated with *Arabidopsis* AGO1 and AGO4 uncovers new AGO functions. Plant J..

[129] Brosseau C., El Oirdi M., Adurogbangba A., Ma X., Moffett P. (2016). Antiviral defense involves AGO4 in an *Arabidopsis*-potexvirus interaction. Mol. Plant-Microbe Interact..

[130] Ye R., Chen Z., Lian B., Rowley M.J., Xia N., Chai J., Li Y., He X.J., Wierzbicki A.T., Qi Y. (2016). A Dicer-independent route for biogenesis of siRNAs that direct DNA methylation in *Arabidopsis*. Mol. Cell.

[131] Raja P., Sanville B.C., Buchmann R.C., Bisaro D.M. (2008). Viral genome methylation as an epigenetic defense against geminiviruses. J. Virol..

[132] Ma X., Nicole M.C., Meteignier L.V., Hong N., Wang G., Moffett P. (2015). Different roles for RNA silencing and RNA processing components in virus recovery and virus-induced gene silencing in plants. J. Exp. Bot..

[133] Fernández-Calvino L., Martínez-Priego L., Szabo E.Z., Guzmán-Benito I., González I., Canto T., Lakatos L., Llave C. (2016). Tobacco rattle virus 16K silencing suppressor binds Argonaute 4 and inhibits formation of RNA silencing complexes. J. Gen. Virol..

[134] Bhattacharjee S., Zamora A., Azhar M.T., Sacco M.A., Lambert L.H., Moffett P. (2009). Virus resistance induced by NB-LRR proteins involves Argonaute 4-dependent translational control. Plant J..

[135] Hamera S., Song X., Su L., Chen X., Fang R. (2012). Cucumber mosaic virus suppressor 2b binds to AGO4-related small RNAs and impairs AGO4 activities. Plant J..

[136] Qin L., Mo N., Zhang Y., Muhammad T., Zhao G., Zhang Y., Liang Y. (2017). *CaRDR1*, an RNA-dependent RNA polymerase plays a positive role in pepper resistance against TMV. Front. Plant Sci..

[137] Yang S.-J., Carter S.A., Cole A.B., Cheng N.-H., Nelson R.S. (2004). A natural variant of a host RNA-dependent RNA polymerase is associated with increased susceptibility to viruses by *Nicotiana benthamiana*. Proc. Natl. Acad. Sci. USA.

[138] Lee W.S., Fu S.F., Li Z., Murphy A.M., Dobson E.A., Garland L., Chaluvadi S.R., Lewsey M.G., Nelson R.S., Carr J.P. (2016). Salicylic acid treatment and expression of an RNA-dependent RNA polymerase 1 transgene inhibit lethal symptoms and meristem invasion during tobacco mosaic virus infection in *Nicotiana benthamiana*. BMC Plant Biol..

[139] Yu D., Fan B., MacFarlane S.A, Chen Z. (2003). Analysis of the involvement of an inducible *Arabidopsis* RNA-dependent RNA polymerase in antiviral defense. Mol. Plant. Microbe Interact..

[140] He J., Dong Z., Jia Z., Wang J., Wang G. (2010). Isolation, expression and functional analysis of a putative RNA-dependent RNA polymerase gene from maize (*Zea mays* L.). Mol. Biol. Rep..

[141] Liao Y.W.K., Liu Y.R., Liang J.Y., Wang W.P., Zhou J., Xia X.J., Zhou Y.H., Yu J.Q., Shi K. (2014). The relationship between the plant-encoded RNA-dependent RNA polymerase 1 and alternative oxidase in tomato basal defense against tobacco mosaic virus. Planta.

[142] Hunter L.J.R., Brockington S.F., Murphy A.M., Pate A.E., Gruden K., MacFarlane S.A., Palukaitis P., Carr J.P. (2016). RNA-dependent RNA polymerase 1 in potato (*Solanum tuberosum*) and its relationship to other plant RNA-dependent RNA polymerases. Sci. Rep..

[143] Xie Z., Fan B., Chen C., Chen Z. (2001). An important role of an inducible RNA-dependent RNA polymerase in plant antiviral defense. Proc. Natl. Acad. Sci. USA.

[144] Qi X., Bao F.S., Xie Z. (2009). Small RNA deep sequencing reveals role for *Arabidopsis thaliana* RNA-dependent RNA polymerases in viral siRNA biogenesis. PLoS ONE.

[145] Verlaan M.G., Hutton S.F., Ibrahem R.M., Kormelink R., Visser R.G.F., Scott J.W., Edwards J.D., Bai Y. (2013). The tomato yellow leaf curl virus resistance genes *Ty-1* and *Ty-3* are allelic and code for DFDGD-class RNA-Dependent RNA polymerases. PLoS Genet..

[146] Caro M., Verlaan M.G., Julián O., Finkers R., Wolters A.M.A., Hutton S.F., Scott J.W., Kormelink R., Visser R.G.F., Díez M.J. (2015). Assessing the genetic variation of *Ty-1* and *Ty-3* alleles conferring resistance to tomato yellow leaf curl virus in a broad tomato germplasm. Mol. Breed..

[147] Verlaan M.G., Szinay D., Hutton S.F., De Jong H., Kormelink R., Visser R.G.F., Scott J.W., Bai Y. (2011). Chromosomal rearrangements between tomato and *Solanum chilense* hamper mapping and breeding of the TYLCV resistance gene *Ty-1*. Plant J..

[148] Butterbach P., Verlaan M.G., Dullemans A., Lohuis D., Visser R.G.F., Bai Y., Kormelink R. (2014). Tomato yellow leaf curl virus resistance by *Ty-1* involves increased cytosine methylation of viral genomes and is compromised by cucumber mosaic virus infection. Proc. Natl. Acad. Sci. USA.

[149] Hutton S.F., Scott J.W., Shekasteband R., Levin I., Lapidot M. (2015). Combinations of *Ty* resistance genes generally provide more effective control against begomoviruses than do single genes. Acta Hortic..

[150] Yamaguchi H., Ohnishi J., Saito A., Ohyama A., Nunome T., Miyatake K., Fukuoka H. (2018). An NB‑LRR gene, TYNBS1, is responsible for resistance mediated by the *Ty‑2 Begomovirus* resistance locus of tomato. Theor. Appl. Genet..

[151] Li Y., Qin L., Zhao J., Muhammad T., Cao H., Li H., Zhang Y., Liang Y. (2017). *SlMAPK3* enhances tolerance to tomato yellow leaf curl virus (TYLCV) by regulating salicylic acid and jasmonic acid signaling in tomato (*Solanum lycopersicum*). PLoS ONE.

[152] Wang X.-B., Wu Q., Ito T., Cillo F., Li W.-X., Chen X., Yu J.-L., Ding S.-W. (2010). RNAi-mediated viral immunity requires amplification of virus-derived siRNAs in *Arabidopsis thaliana*. Proc. Natl. Acad. Sci. USA.

[153] Qiu Y., Wu Y., Zhang Y., Xu W., Wang C., Zhu S. (2018). Profiling of small RNAs derived from cucumber mosaic virus in infected *Nicotiana benthamiana* plants by deep sequencing. Virus Res..

[154] Dalmay T., Horsefield R., Braunstein T.H., Baulcombe D.C. (2001). SDE3 encodes an RNA helicase required for post-transcriptional gene silencing in *Arabidopsis*. EMBO J..

[155] Hernandez-Pinzon I., Yelina N.E., Schwach F., Studholme D.J., Baulcombe D., Dalmay T. (2007). SDE5, the putative homologue of a human mRNA export factor, is required for transgene silencing and accumulation of trans-acting endogenous siRNA. Plant J..

[156] Li F., Wang Y., Zhou X. (2017). SGS3 cooperates with RDR6 in triggering geminivirus-induced gene silencing and in suppressing geminivirus infection in *Nicotiana benthamiana*. Viruses.

[157] Zhang X.P., Liu D.S., Yan T., Fang X.D., Dong K., Xu J., Wang Y., Yu J.L., Wang X.B. (2017). Cucumber mosaic virus coat protein modulates the accumulation of 2b protein and antiviral silencing that causes symptom recovery in planta. PLoS Pathog..

[158] Zhu B., Gao H., Xu G., Wu D., Song S., Jiang H., Zhu S., Qi T., Xie D. (2017). *Arabidopsis* ALA1 and ALA2 mediate RNAi-based antiviral immunity. Front. Plant Sci..

[159] Gao H., Yang M., Yang H., Qin Y., Zhu B., Xu G., Xie C., Wu D., Zhang X., Li W. (2018). *Arabidopsis* ENOR3 regulates RNAi-mediated antiviral defense. J. Genet. Genom..

[160] Jiang L., Qian D., Zheng H., Meng L.Y., Chen J., Le W.J., Zhou T., Zhou Y.J., Wei C.H., Li Y. (2012). RNA-dependent RNA polymerase 6 of rice (*Oryza sativa*) plays role in host defense against negative-strand RNA virus, Rice stripe virus. Virus Res..

[161] Wang M., Li S., Yang H., Gao Z., Wu C., Guo X. (2012). Characterization and functional analysis of *GhRDR6*, a novel RDR6 gene from cotton (*Gossypium hirsutum* L.). Biosci. Rep..

[162] Andika I.B., Sun L., Xiang R., Li J., Chen J. (2013). Root-specific role for *Nicotiana benthamiana* RDR6 in the inhibition of Chinese wheat mosaic virus accumulation at higher temperatures. Mol. Plant-Microbe Interact..

[163] Hong W., Qian D., Sun R., Jiang L., Wang Y., Wei C., Zhang Z., Li Y. (2015). *OsRDR6* plays role in host defense against double-stranded RNA virus, Rice Dwarf Phytoreovirus. Sci. Rep..

[164] Pérez-Cañamás M., Blanco-Pérez M., Forment J., Hernández C. (2017). *Nicotiana benthamiana* plants asymptomatically infected by Pelargonium line pattern virus show unusually high accumulation of viral small RNAs that is neither associated with DCL induction nor RDR6 activity. Virology.

[165] Burgyán J., Havelda Z. (2011). Viral suppressors of RNA silencing. Trends Plant Sci..

[166] Haas G., Azevedo J., Moissiard G., Geldreich A., Himber C., Bureau M., Fukuhara T., Keller M., Voinnet O. (2008). Nuclear import of CaMV P6 is required for infection and suppression of the RNA silencing factor DRB4. EMBO J..

[167] Deleris A., Gallago-Bartolome J., Bao J., Kasschau K.D., Carrington J.C., Voinnet O. (2006). Hierarchical action and inhibition of plant dicer-like proteins in antiviral defense. Science.

[168] Azevedo J., Garcia D., Pontier D., Ohnesorge S., Yu A., Garcia S., Braun L., Bergdoll M., Hakimi M.A., Lagrange T. (2010). Argonaute quenching and global changes in Dicer homeostasis caused by a pathogen-encoded GW repeat protein. Genes Dev..

[169] Giner A., Lakatos L., García-Chapa M., López-Moya J.J., Burgyán J. (2010). Viral protein inhibits RISC activity by Argonaute binding through conserved WG/GW motifs. PLoS Pathog..

[170] Zhang X., Yuan Y., Pei Y., Lin S., Tuschl T., Patel D.J., Chua N. (2006). Cucumber mosaic virus-encoded 2b suppressor inhibits *Arabidopsis* Argonaute1 cleavage activity to counter plant defense. Genes Dev..

[171] Pazhouhandeh M., Dieterle M., Marrocco K., Lechner E., Berry B., Brault V., Hemmer O., Kretsch T., Richards K.E., Genschik P. (2006). F-box-like domain in the polerovirus protein P0 is required for silencing suppressor function. Proc. Natl. Acad. Sci. USA.

[172] Baumberger N., Tsai C.H., Lie M., Havecker E., Baulcombe D.C. (2007). The polerovirus silencing suppressor P0 targets Argonaute proteins for degradation. Curr. Biol..

[173] Bortolamiol D., Pazhouhandeh M., Marrocco K., Genschik P., Ziegler-Graff V. (2007). The polerovirus F box protein P0 targets Argonaute1 to suppress RNA silencing. Curr. Biol..

[174] Kenesi E., Carbonell A., Lózsa R., Vértessy B., Lakatos L. (2017). A viral suppressor of RNA silencing inhibits Argonaute 1 function by precluding target RNA binding to pre-assembled RISC. Nucleic Acids Res..

[175] Silhavy D., Burgyán J. (2004). Effects and side-effects of viral RNA silencing suppressors on short RNAs. Trends Plant Sci..

[176] Martínez-Turiño S., Hernández C. (2009). Inhibition of RNA silencing by the coat protein of Pelargonium flower break virus: Distinctions from closely related suppressors. J. Gen. Virol..

[177] Garcia-Ruiz H., Gabriel Peralta S.M., Harte-Maxwell P.A. (2018). Tomato spotted wilt virus NSs protein supports infection and systemic movement of a potyvirus and is a symptom determinant. Viruses.

[178] Kontra L., Csorba T., Tavazza M., Lucioli A., Tavazza R., Moxon S., Tisza V., Medzihradszky A., Turina M., Burgyán J. (2016). Distinct effects of p19 RNA silencing suppressor on small RNA mediated pathways in plants. PLoS Pathog..

[179] Scholthof H.B., Alvarado V.Y., Vega-Arreguin J.C., Ciomperlik J., Odokonyero D., Brosseau C., Jaubert M., Zamora A., Moffett P. (2011). Identification of an Argonaute for antiviral RNA silencing in *Nicotiana benthamiana*. Plant Physiol..

[180] Odokonyero D., Mendoza M.R., Alvarado V.Y., Zhang J., Wang X., Scholthof H.B. (2015). Transgenic down-regulation of Argonaute 2 expression in *Nicotiana benthamiana* interferes with several layers of antiviral defenses. Virology.

[181] Ruiz-Ruiz S., Soler N., Sánchez-Navarro J., Fagoaga C., López C., Navarro L., Moreno P., Peña L., Flores R. (2013). Citrus tristeza virus p23: Determinants for nucleolar localization and their influence on suppression of RNA silencing and pathogenesis. Mol. Plant-Microbe Interact..

[182] Gómez-Muñoz N., Velázquez K., Vives M.C., Ruiz-Ruiz S., Pina J.A., Flores R., Moreno P., Guerri J. (2017). The resistance of sour orange to Citrus tristeza virus is mediated by both the salycilic acid and the RNA silencing defense pathways. Mol. Plant Pathol..

[183] Varanda C.M.R., Materatski P., Campos M.D., Clara M.I.E., Nolasco G., Félix M.R. (2018). Olive mild mosaic virus coat protein and P6 are suppressors of RNA silencing, and their silencing confers resistance against OMMV. Viruses.

[184] Basu S., Kumar Kushwaha N., Kumar Singh A., Pankaj Sahu P., Vinoth Kumar R., Chakraborty S. (2018). Dynamics of a geminivirus-encoded pre-coat protein and host RNA-dependent RNA polymerase 1 in regulating symptom recovery in tobacco. J. Exp. Bot..

[185] Moissiard G., Parizotto E.A., Himber C., Voinnet O. (2007). Transitivity in *Arabidopsis* can be primed, requires the redundant action of the antiviral Dicer-like 4 and Dicer-like 2, and is compromised by viral-encoded suppressor proteins. RNA.

[186] Glick E., Zrachya A., Levy Y., Mett A., Gidoni D., Belausov E., Citovsky V., Gafni Y. (2008). Interaction with host SGS3 is required for suppression of RNA silencing by tomato yellow leaf curl virus V2 protein. Proc. Natl. Acad. Sci. USA.

[187] Li F., Zhao N., Li Z., Xu X., Wang Y., Yang X., Liu S.S., Wang A., Zhou X. (2017). A calmodulin-like protein suppresses RNA silencing and promotes geminivirus infection by degrading SGS3 via the autophagy pathway in *Nicotiana benthamiana*. PLoS Pathog..

[188] Zhao W., Ji Y., Wu S., Ma X., Li S., Sun F., Cheng Z., Zhou Y., Fan Y. (2018). Single amino acid in V2 encoded by TYLCV is responsible for its self-interaction, aggregates and pathogenicity. Sci. Rep..

[189] Kumar V., Mishra S.K., Rahman J., Taneja J., Sundaresan G., Mishra N.S., Mukherjee S.K. (2015). Mungbean yellow mosaic Indian virus encoded AC2 protein suppresses RNA silencing by inhibiting *Arabidopsis* RDR6 and AGO1 activities. Virology.

[190] Cronin S., Vercho J., Haldeman-Cahil R., Schaad M.C., Carrington J.C. (1995). Long-distance movement factor: A transport function of the potyvirus helper component proteinase. Plant Cell Online.

[191] Senshu H., Yamaji Y., Minato N., Shiraishi T., Maejima K., Hashimoto M., Miura C., Neriya Y., Namba S. (2011). A dual strategy for the suppression of host antiviral silencing: Two distinct suppressors for viral replication and viral movement encoded by potato virus M. J. Virol..

[192] Deng X., Kelloniemi J., Haikonen T., Vuorinen A.L., Elomaa P., Teeri T.H., Valkonen J.P.T. (2013). Modification of Tobacco rattle virus RNA1 to serve as a VIGS vector reveals that the 29K movement protein is an RNA silencing suppressor of the virus. Mol. Plant-Microbe Interact..

[193] Cui H., Wang A. (2016). Plum pox virus 6K1 protein is required for viral replication and targets the viral replication complex at the early stage of infection. J. Virol..

[194] Cui X., Yaghmaiean H., Wu G., Wu X., Chen X., Thorn G., Wang A. (2017). The C-terminal region of the turnip mosaic virus P3 protein is essential for viral infection via targeting P3 to the viral replication complex. Virology.

[195] Wei T., Huang T.-S., McNeil J., Laliberte J.-F., Hong J., Nelson R.S., Wang A. (2010). Sequential recruitment of the endoplasmic reticulum and chloroplasts for plant potyvirus replication. J. Virol..

[196] Wei T., Zhang C., Hou X., Sanfaçon H., Wang A. (2013). The SNARE protein Syp71 is essential for turnip mosaic virus infection by mediating fusion of virus-induced vesicles with chloroplasts. PLoS Pathog..

[197] Endres M.W., Gregory B.D., Gao Z., Foreman A.W., Mlotshwa S., Ge X., Pruss G.J., Ecker J.R., Bowman L.H., Vance V. (2010). Two plant viral suppressors of silencing require the ethylene-inducible host transcription factor RAV2 to block RNA silencing. PLoS Pathog..

[198] Li F., Wang A. (2018). RNA decay is an antiviral defense in plants that is counteracted by viral RNA silencing suppressors. PLoS Pathog..

[199] Li F., Huang C., Li Z., Zhou X. (2014). Suppression of RNA silencing by a plant DNA virus satellite requires a host calmodulin-like protein to repress RDR6 expression. PLoS Pathog..

[200] Yong Chung H., Lacatus G., Sunter G. (2014). Geminivirus AL2 protein induces expression of, and interacts with, a calmodulin-like gene, an endogenous regulator of gene silencing. Virology.

[201] Segers G.C., Zhang X., Deng F., Sun Q., Nuss D.L. (2007). Evidence that RNA silencing functions as an antiviral defense mechanism in fungi. Proc. Natl. Acad. Sci. USA.

[202] Chang S.-S., Zhang Z., Liu Y. (2012). RNA interference pathways in fungi: Mechanisms and functions. Annu. Rev. Microbiol..

[203] Chiba S., Lin Y.-H., Kondo H., Kanematsu S., Suzuki N. (2013). A novel victorivirus from a phytopathogenic fungus, *Rosellinia necatrix*, is infectious as particles and targeted by RNA silencing. J. Virol..

[204] Qian X., Hamid F.M., El Sahili A., Darwis D.A., Wong Y.H., Bhushan S., Makeyev E.V., Lescar J. (2016). Functional evolution in orthologous cell-encoded RNA-dependent RNA polymerases. J. Biol. Chem..

[205] Alexander W.G., Raju N.B., Xiao H., Hammond T.M., Perdue T.D., Metzenberg R.L., Pukkila P.J., Shiu P.K.T. (2008). DCL-1 colocalizes with other components of the MSUD machinery and is required for silencing. Fungal Genet. Biol..

[206] Sun Q., Choi G.H., Nuss D.L. (2009). A single Argonaute gene is required for induction of RNA silencing antiviral defense and promotes viral RNA recombination. Proc. Natl. Acad. Sci. USA.

[207] Meng H., Wang Z., Wang Y., Zhu H., Huanga B. (2017). Dicer and Argonaute genes involved in RNA interference in the entomopathogenic fungus *Metarhizium robertsii*. Appl. Environ. Microbiol..

[208] Nguyen Q., Iritani A., Ohkita S., Vu B.V., Yokoya K., Matsubara A., Ikeda K., Suzuki N., Nakayashiki H. (2018). A fungal Argonaute interferes with RNA interference. Nucleic Acids Res..

[209] Weiberg A., Wang M., Lin F.M., Zhao H., Zhang Z., Kaloshian I., Huang H.D., Jin H. (2013). Fungal small RNAs suppress plant immunity by hijacking host RNA interference pathways. Science.

[210] Feng H., Xu M., Liu Y., Gao X., Yin Z., Voegele R.T., Huang L. (2017). The distinct roles of Argonaute protein 2 in the growth, stress responses and pathogenicity of the apple tree canker pathogen. For. Pathol..

[211] Campo S., Gilbert K.B., Carrington J.C. (2016). Small RNA-based antiviral defense in the phytopathogenic fungus *Colletotrichum higginsianum*. PLoS Pathog..

[212] Wang Q., An B., Hou X., Guo Y., Luo H., He C. (2018). Dicer-like proteins regulate the growth, conidiation, and pathogenicity of *Colletotrichum gloeosporioides* from *Hevea brasiliensis*. Front. Microbiol..

[213] Ellendorff U., Fradin E.F., De Jonge R., Thomma B.P.H.J. (2009). RNA silencing is required for *Arabidopsis* defence against *Verticillium* wilt disease. J. Exp. Bot..

[214] Cao J.Y., Xu Y.P., Zhao L., Li S.S., Cai X.Z. (2016). Tight regulation of the interaction between *Brassica napus* and *Sclerotinia sclerotiorum* at the microRNA level. Plant Mol. Biol..

[215] Guo N., Zhao J., Yan Q., Huang J., Ma H., Rajput N.A., Jiang H., Xing H., Dou D. (2018). Resistance to *Phytophthora* pathogens is dependent on gene silencing pathways in plants. J. Phytopathol..

[216] Zhang D., Liu M., Tang M., Dong B., Wu D., Zhang Z., Zhou B. (2015). Repression of microRNA biogenesis by silencing of *OsDCL1* activates the basal resistance to *Magnaporthe oryzae* in rice. Plant Sci..

[217] Liu B., Li P., Li X., Liu C., Cao S., Chu C., Cao X. (2005). Loss of function of *OsDCL1* affects microRNA accumulation and causes developmental defects in rice. Plant Physiol..

[218] Shen D., Suhrkamp I., Wang Y., Liu S., Menkhaus J., Verreet J.A., Fan L., Cai D. (2014). Identification and characterization of microRNAs in oilseed rape (*Brassica napus*) responsive to infection with the pathogenic fungus *Verticillium longisporum* using Brassica AA (*Brassica rapa*) and CC (*Brassica oleracea*) as refer. New Phytol..

[219] Várallyay É., Válóczi A., Ágyi Á., Burgyán J., Havelda Z. (2010). Plant virus-mediated induction of miR168 is associated with repression of Argonaute1 accumulation. EMBO J..

[220] Ouyang S., Park G., Atamian H.S., Han C.S., Stajich J.E., Kaloshian I., Borkovich K.A. (2014). MicroRNAs suppress NB domain genes in tomato that confer resistance to *Fusarium oxysporum*. PLoS Pathog..

[221] Jones-Rhoades M.W., Bartel D.P., Bartel B. (2006). MicroRNAs and their regulatory roles in plants. Annu. Rev. Plant Biol..

[222] Zhu Q.H., Fan L., Liu Y., Xu H., Llewellyn D., Wilson I. (2013). miR482 regulation of NBS-LRR defense genes during fungal pathogen infection in cotton. PLoS ONE.

[223] Campo S., Peris-Peris C., Siré C., Moreno A.B., Donaire L., Zytnicki M., Notredame C., Llave C., San Segundo B. (2013). Identification of a novel microRNA (miRNA) from rice that targets an alternatively spliced transcript of the Nramp6 (Natural resistance-associated macrophage protein 6) gene involved in pathogen resistance. New Phytol..

[224] Li Y., Lu Y.-G., Shi Y., Wu L., Xu Y.-J., Huang F., Guo X.-Y., Zhang Y., Fan J., Zhao J.-Q. (2014). Multiple rice microRNAs are involved in immunity against the blast fungus *Magnaporthe oryzae*. Plant Physiol..

[225] Chen M., Cao Z. (2015). Genome-wide expression profiling of microRNAs in poplar upon infection with the foliar rust fungus *Melampsora larici-populina*. BMC Genom..

[226] Chen L., Luan Y., Zhai J. (2015). Sp-miR396a-5p acts as a stress-responsive genes regulator by conferring tolerance to abiotic stresses and susceptibility to *Phytophthora nicotianae* infection in transgenic tobacco. Plant Cell Rep..

[227] Cai Q., Qiao L., Wang M., He B., Lin F.M., Palmquist J., Huang S.D., Jin H. (2018). Plants send small RNAs in extracellular vesicles to fungal pathogen to silence virulence genes. Science.

[228] Zhang T., Zhao Y.L., Zhao J.H., Wang S., Jin Y., Chen Z.Q., Fang Y.Y., Hua C.L., Ding S.W., Guo H.S. (2016). Cotton plants export microRNAs to inhibit virulence gene expression in a fungal pathogen. Nat. Plants.

[229] Rutter B.D., Innes R.W. (2017). Extracellular vesicles isolated from the leaf apoplast carry stress-response proteins. Plant Physiol..

[230] Wang M., Weiberg A., Dellota E., Yamane D., Jin H. (2017). Botrytis small RNA *Bc-siR37* suppresses plant defense genes by cross-kingdom RNAi. RNA Biol..

[231] Andrade C.M., Tinoco M.L.P., Rieth A.F., Maia F.C.O., Aragão F.J.L. (2016). Host-induced gene silencing in the necrotrophic fungal pathogen *Sclerotinia sclerotiorum*. Plant Pathol..

[232] Cheng W., Song X.S., Li H.P., Cao L.H., Sun K., Qiu X.L., Xu Y.B., Yang P., Huang T., Zhang J.B. (2015). Host-induced gene silencing of an essential chitin synthase gene confers durable resistance to *Fusarium* head blight and seedling blight in wheat. Plant Biotechnol. J..

[233] Song Y., Thomma B.P.H.J. (2016). Host-induced gene silencing compromises *Verticillium* wilt in tomato and *Arabidopsis*. Mol. Plant Pathol..

[234] Chen W., Kastner C., Nowara D., Oliveira-Garcia E., Rutten T., Zhao Y., Deising H.B., Kumlehn J., Schweizer P. (2016). Host-induced silencing of *Fusarium culmorum* genes protects wheat from infection. J. Exp. Bot..

[235] Panwar V., McCallum B., Bakkeren G. (2013). Host-induced gene silencing of wheat leaf rust fungus *Puccinia triticina* pathogenicity genes mediated by the *Barley stripe mosaic virus*. Plant Mol. Biol..

[236] Hu Z., Parekh U., Maruta N., Trusov Y., Botella J.R. (2015). Down-regulation of *Fusarium oxysporum* endogenous genes by host-delivered RNA interference enhances disease resistance. Front. Chem..

[237] Yin C., Downey S.I., Klages-Mundt N.L., Ramachandran S., Chen X., Szabo L.J., Pumphrey M., Hulbert S.H. (2015). Identification of promising host-induced silencing targets among genes preferentially transcribed in haustoria of *Puccinia*. BMC Genom..

[238] Zhou B., Bailey A., Niblett C.L., Qu R. (2016). Control of brown patch (*Rhizoctonia solani*) in tall fescue (*Festuca arundinacea* Schreb.) by host induced gene silencing. Plant Cell Rep..

[239] Quiñones B., Dulla G., Lindow S.E. (2005). Quorum sensing regulates exopolysaccharide production, motility, and virulence in *Pseudomonas syringae*. Mol. Plant-Microbe Interact..

[240] Aslam S.N., Newman M.A., Erbs G., Morrissey K.L., Chinchilla D., Boller T., Jensen T.T., De Castro C., Ierano T., Molinaro A. (2008). Bacterial polysaccharides suppress induced innate immunity by calcium chelation. Curr. Biol..

[241] Block A., Li G., Fu Z.Q., Alfano J.R. (2008). Phytopathogen type III effector weaponry and their plant targets. Curr. Opin. Plant Biol..

[242] Fones H., Preston G.M. (2013). The impact of transition metals on bacterial plant disease. FEMS Microbiol. Rev..

[243] Kunkel B.N., Harper C.P. (2018). The roles of auxin during interactions between bacterial plant pathogens and their hosts. J. Exp. Bot..

[244] Ronald P., Joe A. (2018). Molecular mimicry modulates plant host responses to pathogens. Ann. Bot..

[245] Pumplin N., Voinnet O. (2013). RNA silencing suppression by plant pathogens: Defence, counter-defence and counter-counter-defence. Nat. Rev. Microbiol..

[246] Staiger D., Korneli C., Lummer M., Navarro L. (2013). Emerging role for RNA-based regulation in plant immunity. New Phytol..

[247] Nobori T., Velásquez A.C., Wu J., Kvitko B.H., Kremer J.M., Wang Y., He S.Y., Tsuda K. (2018). Transcriptome landscape of a bacterial pathogen under plant immunity. Proc. Natl. Acad. Sci. USA.

[248] Navarro L., Jay F., Nomura K., He S.Y., Voinnet O. (2008). Suppression of the microRNA pathway by bacterial effector proteins. Science.

[249] Dunoyer P., Himber C., Voinnet O. (2006). Induction, suppression and requirement of RNA silencing pathways in virulent *Agrobacterium tumefaciens* infections. Nat. Genet..

[250] Katiyar-agarwal S., Gao S., Vivian-smith A., Jin H. (2007). A novel class of bacteria-induced small RNAs in *Arabidopsis*. GENES Dev..

[251] Li Y., Zhang Q., Zhang J., Wu L., Qi Y., Zhou J.M. (2010). Identification of microRNAs involved in pathogen-associated molecular pattern-triggered plant innate immunity. Plant Physiol..

[252] Liu C., Xin Y., Xu L., Cai Z., Xue Y., Liu Y., Xie D., Liu Y., Qi Y. (2018). *Arabidopsis* Argonaute 1 binds chromatin to promote gene transcription in response to hormones and stresses. Dev. Cell.

[253] Navarro L., Dunoyer P., Jay F., Arnold B., Dharmasiri N., Estelle M., Voinnet O., Jones J.D.G. (2006). A plant miRNA contributes to antibacterial resistance by repressing auxin signaling. Science.

[254] Zhang X., Zhao H., Gao S., Wang W.C., Katiyar-Agarwal S., Huang H.D., Raikhel N., Jin H. (2011). *Arabidopsis* Argonaute 2 regulates innate immunity via miRNA393*-mediated silencing of a golgi-localized SNARE gene, MEMB12. Mol. Cell.

[255] Xia R., Xu J., Meyers B.C. (2017). The emergence, evolution, and diversification of the miR390-TAS3-ARF pathway in land plants. Plant Cell.

[256] Guilfoyle T.J., Hagen G. (2007). Auxin response factors. Curr. Opin. Plant Biol..

[257] Agorio A., Vera P. (2007). Argonaute 4 is required for resistance to *Pseudomonas syringae* in *Arabidopsis*. Plant Cell Online.

[258] Yu A., Lepere G., Jay F., Wang J., Bapaume L., Wang Y., Abraham A.-L., Penterman J., Fischer R.L., Voinnet O. (2013). Dynamics and biological relevance of DNA demethylation in *Arabidopsis* antibacterial defense. Proc. Natl. Acad. Sci. USA.

[259] Katiyar-Agarwal S., Morgan R., Dahlbeck D., Borsani O., Villegas A., Zhu J.-K., Staskawicz B.J., Jin H. (2006). A pathogen-inducible endogenous siRNA in plant immunity. Proc. Natl. Acad. Sci. USA.

[260] Wagh S.G., Alam M.M., Kobayashi K., Yaeno T., Yamaoka N., Toriba T., Hirano H.Y., Nishiguchi M. (2016). Analysis of rice RNA-dependent RNA polymerase 6 (*OsRDR6*) gene in response to viral, bacterial and fungal pathogens. J. Gen. Plant Pathol..

[261] Zhai J., Jeong D.H., de Paoli E., Park S., Rosen B.D., Li Y., González A.J., Yan Z., Kitto S.L., Grusak M.A. (2011). MicroRNAs as master regulators of the plant NB-LRR defense gene family via the production of phased, trans-acting siRNAs. Genes Dev..

[262] Li F., Pignatta D., Bendix C., Brunkard J.O., Cohn M.M., Tung J., Sun H. (2012). MicroRNA regulation of plant innate immune receptors. Proc. Natl. Acad. Sci. USA.

[263] Shivaprasad P.V., Chen H.-M., Patel K., Bond D.M., Santos B.A.C.M., Baulcombe D.C. (2012). A microRNA superfamily regulates nucleotide binding site-leucine-rich repeats and other mRNAs. Plant Cell.

[264] Fei Q., Yu Y., Liu L., Zhang Y., Baldrich P., Dai Q., Chen X., Meyers B.C. (2018). Biogenesis of a 22-nt microRNA in Phaseoleae species by precursor-programmed uridylation. Proc. Natl. Acad. Sci. USA.

[265] Niu D., Xia J., Jiang C., Qi B., Ling X., Lin S., Zhang W., Guo J., Jin H., Zhao H. (2016). *Bacillus cereus* AR156 primes induced systemic resistance by suppressing miR825/825* and activating defense-related genes in *Arabidopsis*. J. Integr. Plant Biol..

[266] Boccara M., Sarazin A., Thiébeauld O., Jay F., Voinnet O., Navarro L., Colot V. (2014). The *Arabidopsis* miR472-RDR6 silencing pathway modulates PAMP- and effector-triggered immunity through the post-transcriptional control of disease resistance genes. PLoS Pathog..

[267] Kurihara Y., Watanabe Y. (2004). *Arabidopsis* micro-RNA biogenesis through Dicer-like 1 protein functions. Proc. Natl. Acad. Sci. USA.

[268] Parent J.S., Bouteiller N., Elmayan T., Vaucheret H. (2015). Respective contributions of *Arabidopsis* DCL2 and DCL4 to RNA silencing. Plant J..

[269] Fusaro A.F., Matthew L., Smith N.A., Curtin S.J., Dedic-Hagan J., Ellacott G.A., Watson J.M., Wang M.B., Brosnan C., Carroll B.J. (2006). RNA interference-inducing hairpin RNAs in plants act through the viral defence pathway. EMBO Rep..

[270] Yang L., Wu G., Poethig R.S. (2012). Mutations in the GW-repeat protein SUO reveal a developmental function for microRNA-mediated translational repression in *Arabidopsis*. Proc. Natl. Acad. Sci. USA.

[271] Fátyol K., Ludman M., Burgyán J. (2016). Functional dissection of a plant Argonaute. Nucleic Acids Res..

[272] Gao Z., Liu H.L., Daxinger L., Pontes O., He X., Qian W., Lin H., Xie M., Lorkovic Z.J., Zhang S. (2010). An RNA polymerase II-and AGO4-associated protein acts in RNA-directed DNA methylation. Nature.

[273] Minoia S., Carbonell A., Di Serio F., Gisel A., Carrington J.C., Navarro B., Flores R. (2014). Specific Argonautes selectively bind small RNAs derived from Potato spindle tuber viroid and attenuate viroid accumulation in vivo. J. Virol..

[274] Schwach F., Vaistij F.E., Jones L., Baulcombe D.C. (2005). An RNA-dependent RNA polymerase prevents meristem invasion by Potato virus X and is required for the activity but not the production of a systemic silencing signal. Plant Physiol..

[275] Yang Z., Ebright Y.W., Yu B., Chen X. (2006). HEN1 recognizes 21–24 nt small RNA duplexes and deposits a methyl group onto the 2′ OH of the 3′ terminal nucleotide. Nucleic Acids Res..

[276] Iki T., Yoshikawa M., Nishikiori M., Jaudal M.C., Matsumoto-Yokoyama E., Mitsuhara I., Meshi T., Ishikawa M. (2010). In vitro assembly of plant RNA-induced silencing complexes facilitated by molecular chaperone HSP90. Mol. Cell.

[277] Yoshikawa M., Iki T., Tsutsui Y., Miyashita K., Poethig R.S., Habu Y., Ishikawa M. (2013). 3′-fragment of miR173-programmed RISC-cleaved RNA is protected from degradation in a complex with RISC and SGS3. Proc. Natl. Acad. Sci. USA.

